# New K50R mutant mouse models reveal impaired hypusination of eif5a2 with alterations in cell metabolite landscape

**DOI:** 10.1242/bio.059647

**Published:** 2023-03-21

**Authors:** Chad R. Schultz, Ryan D. Sheldon, Huirong Xie, Elena Y. Demireva, Katie L. Uhl, Dalen W. Agnew, Dirk Geerts, André S. Bachmann

**Affiliations:** ^1^ Department of Pediatrics and Human Development, College of Human Medicine, Michigan State University, Grand Rapids, MI 49503, USA; ^2^ Core Technologies and Services, Mass Spectrometry Core, Van Andel Institute, Grand Rapids, MI 49503, USA; ^3^ Transgenic and Genome Editing Facility, Institute for Quantitative Health Science and Engineering, Research Technology Support Facility, Michigan State University, East Lansing, MI 48824, USA; ^4^ Department of Pathobiology & Diagnostic Investigation, College of Veterinary Medicine, Michigan State University, East Lansing, MI 48824, USA; ^5^ Department of Hematology, Amsterdam University Medical Center, Location VUMC, 1081 HV Amsterdam, The Netherlands

**Keywords:** CRISPR, EIF5A1, EIF5A2, Hypusine, K50R mutation, Metabolomics, Mouse models

## Abstract

The eukaryotic translation initiation factor 5A1 (eIF5A1) and 5A2 (eIF5A2) are important proteins in a variety of physiological and pathophysiological processes and their function has been linked to neurodevelopmental disorders, cancer, and viral infections. Here, we report two new genome-edited mouse models, generated using a CRISPR-Cas9 approach, in which the amino acid residue lysine 50 is replaced with arginine 50 (K50R) in eIF5A1 or in the closely related eIF5A2 protein. This mutation prevents the spermidine-dependent post-translational formation of hypusine, a unique lysine derivative that is necessary for activation of eIF5A1 and eIF5A2. Mouse brain lysates from homozygous eif5a2-K50R mutant mice (*eif5a2^K50R/K50R^*) confirmed the absence of hypusine formation of eIF5A2, and metabolomic analysis of primary mouse dermal fibroblasts revealed significant alterations in the metabolite landscape compared to controls including increased levels of tryptophan, kyrunenine, pyridoxine, nicotinamide adenine dinucleotide, riboflavin, flavin adenine dinucleotide, pantothenate, and coenzyme A. Further supported by new publicly available bioinformatics data, these new mouse models represent excellent *in vivo* models to study hypusine-dependent biological processes, hypusination-related disorders caused by *eIF5A1* and *eIF5A2* gene aberrations or mRNA expression dysregulation, as well as several major human cancer types and potential therapies.

## Teaser

**Summary:** We present new genome-edited mouse models in which the amino acid residue lysine 50 is replaced with arginine 50 (K50R) in either eIF5A1 or eIF5A2 proteins. This mutation prevents the spermidine-dependent post-translational formation of hypusine, a unique lysine derivative that is necessary for activation of eIF5A1 and eIF5A2.

## INTRODUCTION

**Fig. 1.**
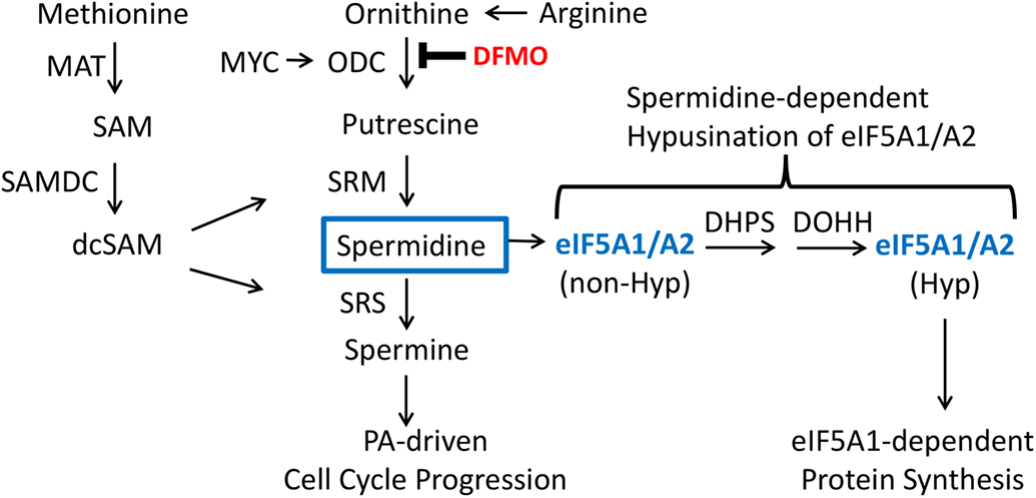
**The polyamine-hypusine circuitry.** ODC is a rate-limiting enzyme in the biosynthesis of polyamines (putrescine, spermidine, spermine), which are dysregulated in polyamine-associated neurodevelopmental disorders and many cancers. ODC is a *bona fide* target of MYC. DFMO (also known as eflornithine, an anti-protozoan drug) is an irreversible ODC inhibitor that blocks polyamine synthesis. Methionine provides dcSAM, which is needed for the synthesis of spermidine and spermine. eIF5A1/A2 hypusination strictly depends on the presence of spermidine, the substrate in the formation of the unique amino acid hypusine, by post-translational modification of lysine (at K50) through a two-step enzymatic reaction involving DHPS and DOHH. ODC, ornithine decarboxylase; MAT methionine adenosyltransferase; SAM, *S*-adenosylmethionine; SAMDC (also AMD1 or AdoMetDC), *S*-adensylmethionine decarboxylase; dcSAM, decarboxylasted *S*-adenosylmethionine; DPHS, deoxyhypusine synthase; DOHH, deoxyhypusine hydroxylase; SRM, spermidine synthase; SRS, spermine synthase; Hyp, hypusinated; DFMO, difluoromethylornithine.

The eukaryotic translation initiation factor 5A1 (eIF5A1, also called eIF5A) and eukaryotic translation initiation factor 5A2 (eIF5A2) are the only known human proteins to feature the unique amino acid hypusine, a post-translationally modified, conserved lysine residue at amino acid position 50 (K50). Both eIF5A1 and eIF5A2 are hypusinated through two-step enzymatic reactions involving deoxyhypusine synthase (DHPS) and deoxyhypusine hydroxylase (DOHH) (Fig. 1) (Park et al., 2006, 2022; Park and Wolff, 2018). DHPS catalyzes the transfer of the 4-aminobutyl moiety from the polyamine spermidine to K50 and forms the intermediate deoxyhypusine, which subsequently is hydroxylated by DOHH to synthesize hypusine. Spermidine is the sole substrate for the post-translational formation of hypusine and, therefore, essential for eIF5A1 and eIF5A2 functionality (Park et al., 1981). The three major polyamines are putrescine, spermidine, and spermine. Ornithine is converted by ornithine decarboxylase (ODC) to putrescine, the precursor of spermidine and spermine (Fig. 1) (Nakanishi and Cleveland, 2016).

The two evolutionarily conserved human proteins eIF5A1 and eIF5A2 are 84% identical and 94% similar (Jenkins et al., 2001). They play a critical role in the synthesis of peptide bonds between consecutive proline residues and resolve ribosomal stalling. Initially thought to be a general translation initiation factor, eIF5A1 was later found to promote translation elongation and to be especially important for synthesis of proteins containing poly-proline motifs (Gutierrez et al., 2013; Landau et al., 2010; Nakanishi and Cleveland, 2016; Park, 2006, 1981, 2010; Saini et al., 2009; Zanelli and Valentini, 2007). More recently, reports have shown that eIF5A1 also promotes translation elongation of non-proline tripeptide sequences and is involved in translation termination (Pelechano and Alepuz, 2017; Schuller et al., 2017). Strikingly, eIF5A1 is pivotal for neuronal growth and cell survival, thus suggesting a role in neurodevelopmental disorders.

Hypusine modification of eIF5A1 and eIF5A2 at K50 is essential for protein activity; it supports eIF5A-mediated translation elongation through peptide bond formation at polyproline and ribosome-pausing sites and increases translation termination by activating peptide release (Park et al., 2022, 2010; Park and Wolff, 2018). eIF5A proteins are important in physiological and pathophysiological processes including protein synthesis and biomass production in cancer (Bandino et al., 2014; Coni et al., 2020; Kim et al., 2022; Liu et al., 2016; Mathews and Hershey, 2015; Nakanishi and Cleveland, 2016; Schultz et al., 2018; Wang et al., 2013; Wei et al., 2014), embryonic and neuronal development, Alzheimer's disease (Smeltzer et al., 2021), and viral replication (Ebola, Zika, Dengue, other) (Farache et al., 2022; Olsen et al., 2018; Singh et al., 2022). Therefore, the generation of new *in vivo* models to produce more insights into hypusine-dependent mechanisms will provide insight into several diseases and offer clues to the development of new therapies.

Importantly, eIF5A1 mutations were recently found to cause the autosomal dominant craniofacial-neurodevelopmental disorder Faundes-Banka syndrome (FABAS), but the pathogenesis of FABAS remains poorly understood [Online Mendelian Inheritance in Man (OMIM) database number 619376] (Faundes et al., 2021; Park et al., 2022). FABAS is characterized by variable combinations of developmental delay and microcephaly as well as micrognathia and other dysmorphic features. To our knowledge, eIF5A2 mutations causing protein changes in human have not been identified. While eIF5A1 is ubiquitously and quite highly expressed, eIF5A2 under physiological conditions is only expressed at lower levels in a few human tissues including brain, lung, prostate, and testis (Jenkins et al., 2001; Pallmann et al., 2015). However, eIF5A2 overexpression plays an important role in a variety of cancers, with particularly high expression in colorectal adenocarcinoma (Bai et al., 2018; Jenkins et al., 2001; Liu et al., 2016; Nakanishi and Cleveland, 2016; Wang et al., 2013; Wei et al., 2014).

Several mouse and zebrafish models to study eif5a1, eif5a2, Dhps, and Dohh are available and revealed that homozygous null-mutant mice were embryonic lethal, while heterozygous mice were viable and fertile (Faundes et al., 2021; Kar et al., 2021; Mastracci et al., 2015; Nishimura et al., 2012; Pallmann et al., 2015; Sievert et al., 2014). Mutant mice with a temporal or region-specific knockout of eif5a1 or Dhps in the forebrain exhibit impairment in growth, lifespan, brain development, and cognitive functions (Kar et al., 2021) and are similar to phenotypes described in humans (Ziegler et al., 2022). These models will be useful for the development of future treatments against neurodevelopmental disorders caused by variants of these genes.

To generate additional *in vivo* models that allow us to study specifically how hypusination controls the biological functions of eif5a1 and eif5a2 in development and disease, we mutated the K50 residue in both genes and generated K50R mutant mice. Unlike the existing knockout models that do not synthesize eif5a1 or eif5a2 proteins, our mice synthesize full length eif5a1 or eif5a2 proteins that cannot be hypusinated through post-translational modification. The new CRISPR-generated *eif5a1^K50R/+^* and *eif5a2^K50R/K50R^* mouse strains that express full-length K50R mutant protein are now available at the Mutant Mouse Resource and Research Centers (MMRRC). With our primary research focus on eIF5A2, we further analyzed the *eif5a2^K50R/K50R^* mouse strain and created a metabolomic landscape profile comparing primary dermal mouse fibroblasts of wild-type mice with our homozygous *eif5a2^K50R/K50R^* mice. Both models are available to the research community and will be useful *in vivo* tools for future studies to understand the roles of eIF5A1 and eIF5A2 hypusination in human biology and the pathophysiology of various diseases including eIF5A1-associated neurodevelopmental disorders like FABAS, cancer, and infectious diseases.

## RESULTS

**Fig. 2.**
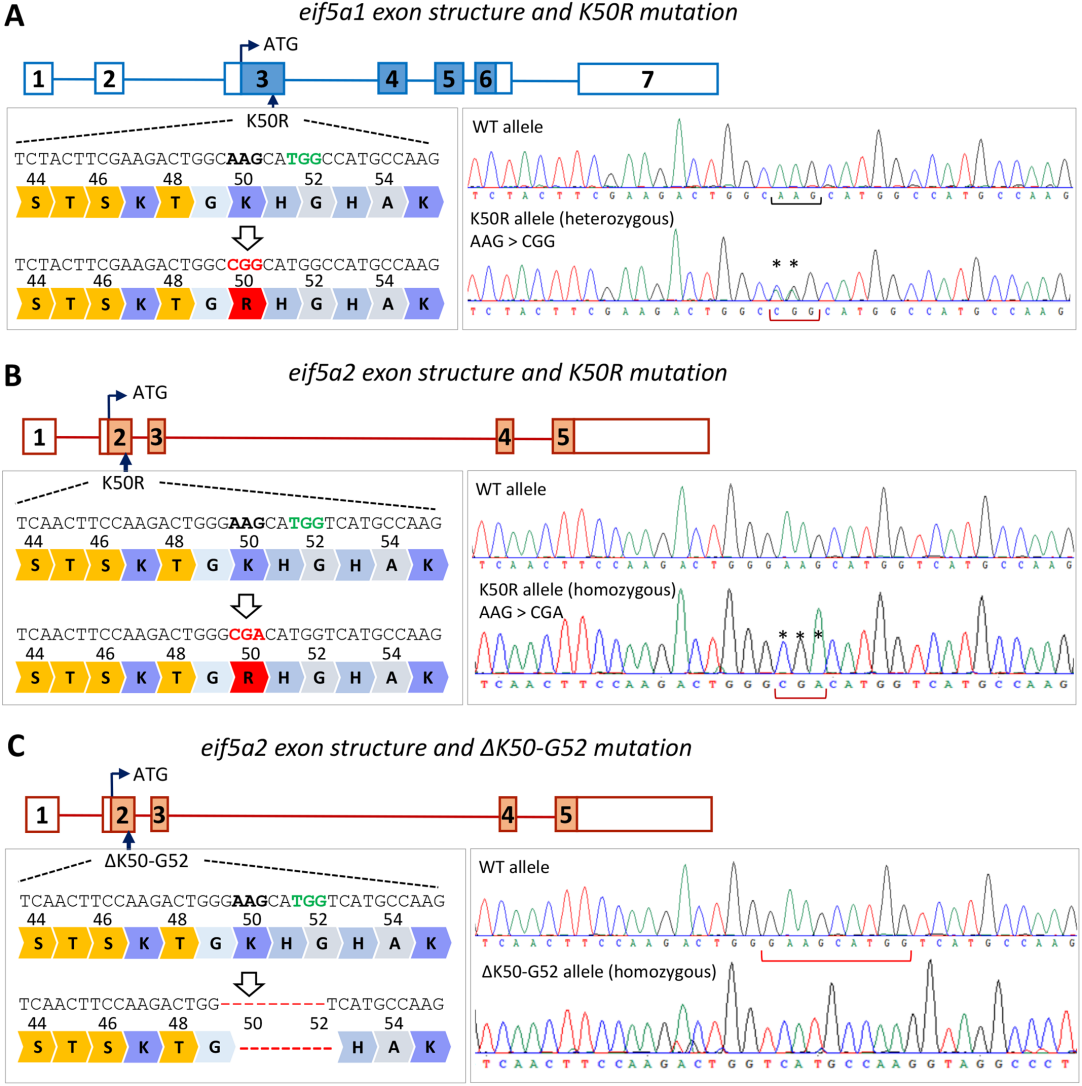
**Generation of *eif5a1-K50R* and *eif5a2-K50R* mutant mice.** Targeting strategy and sequencing results for *eif5a1-K50R* (A), *eif5a2-K50R* (B) and *eif5a2-ΔK50-G52* (C) mouse lines. In schematics of exon structure, filled rectangles denote coding exons, and empty exons correspond to untranslated regions. Relative position of ATG (start codon) and K50R codon are indicated, with target site at K50 magnified to show the position of flanking amino acids and DNA sequence (left panels). DNA sequence highlighted in bold – K50 codon, red – R50 mutation, green – PAM of gRNA used for CRISPR targeting. Sanger sequencing chromatograms (right panels) of the wild-type allele and K50R-mutated allele in a heterozygous (A) and homozygous (B and C) format are labeled with asterisks to denote mutated nucleotides, and with brackets marking K50R codon position. PAM, protospacer adjacent motif; WT, wild-type.

In this study, we generated two new eif5a1 and eif5a2 mutant mouse models that will be available to the research community for *in vivo* studies on hypusine-dependent biological processes and diseases. CRISPR-Cas9 editing was used to generate mice with a single amino acid substitution (K50R) in the eif5a1 or eif5a2 proteins (Fig. 2). In addition, for eif5a2, a 9-bp deletion allele that removes the hypusinated site plus 2 adjacent amino acids (ΔK50-G52) was generated (Table 1). After multiple rounds of back crossing, we were able to establish heterozygous *eif5a1-K50R* (*eif5a1^K50R/+^*) and homozygous eif5a2-K50R (*eif5a2^K50R/K50R^*) mouse lines. Homozygous *eif5a1^K50R/K50R^* animals were never recovered from heterozygous crossings, pointing to a potential embryonic lethality for homozygous animals (Table 2). Heterozygous *eif5a1^K50R/+^* females had considerably smaller litter sizes (2.33 pups/litter) and from six recorded litters only produced six pups that reached weaning age (Table 2). Three pups were wild-type (two female, one male) and three pups were heterozygous (two female, one male). Three of the six litters resulted in neglect and death of eight pups. Five of the eight dead pups were genotyped (two wild-type and three heterozygous). The dead pups were 1-2 days old only, and their sex was not determined.

**Table 1.**
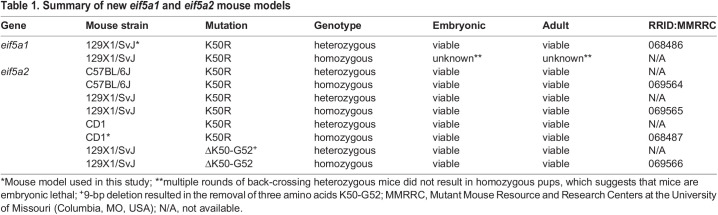
Summary of new *eif5a1* and *eif5a2* mouse models

**Table 2.**
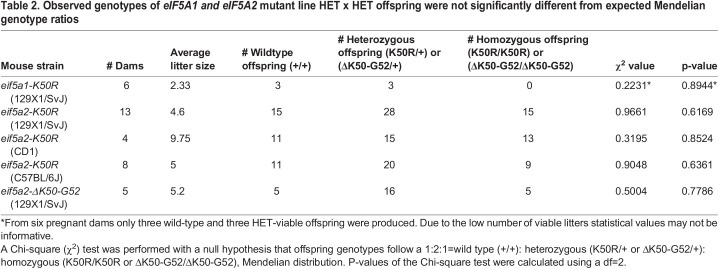
Observed genotypes of *eIF5A1 and eIF5A2* mutant line HET x HET offspring were not significantly different from expected Mendelian genotype ratios

**Fig. 3.**
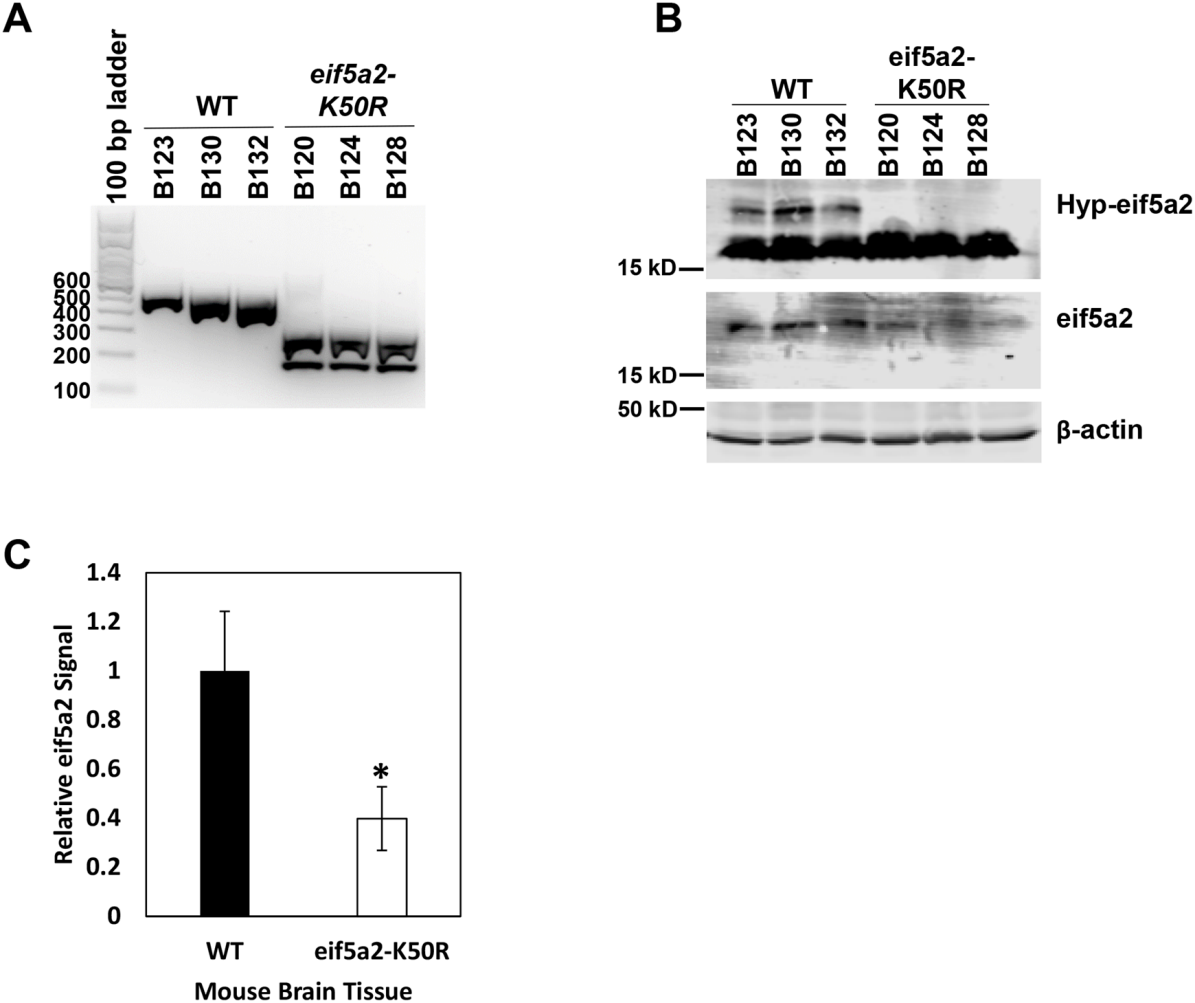
**Validation of the genotype and hypusination status of the *eif5a2-K50R* mouse model.** (A) Mice were genotyped to confirm the *eif5a2-K50R* mutation using mouse tail DNA preps. The O208/O209 primer pair was used for PCR resulting in a 503 bp product, which was digested using *Pfl*FI. *eif5a2-K50R* mutant PCR product is cut into two bands, while the wild-type product is not digested. (B) Protein lysates from brain tissue of the same mice were used to detect hypusinated eif5a2 levels. Brain tissue was used because of its high endogenous eif5a2 expression. As expected, *eif5a2^K50R/K50R^* mice lack hypusinated eif5a2. (C) Quantification of eif5a2 levels relative to β-actin in (B). * indicates a statistically significant difference in eif5a2 protein levels in wild-type and *eif5a2-K50R* mouse brain lysates (*P*=0.0194). WT, wild-type.

In contrast, homozygous male and female *eif5a2^K50R/K50R^* mice were viable and fertile and, therefore, were selected for further studies (Tables 1 and 2). Western blot analysis was used to examine eif5a2 protein expression and impaired hypusination in brain tissues of *eif5a2^K50R/K50R^* mice using eIF5A2 protein- and hypusine-specific antibodies. Of note, the hypusine-specific antibody can identify the hypusine modification as well as the deoxyhypusine intermediate. As shown in Fig. 3B, hypusine was absent in brain tissues of *eif5a2^K50R/K50R^* mice and the total amount of eif5a2 protein was reduced (Fig. 3C) compared to wild-type mice. In addition, we confirmed that the indicated band shown in Fig. 3B is indeed hypusinated eif5a2 (Fig. S1). Whole-body to organ-weight ratios between *eif5a2^K50R/K50R^* and wild-type mice did not reveal any significant differences (Fig. S2) and major organs including spleen, lung, liver, heart, adrenal gland, thymus, ovary, testicle, uterus, kidney, and brain of *eif5a2^K50R/K50R^* and wild-type mice were compared by Hematoxylin and Eosin staining and did not show any differences (Fig. S3). This suggests that the eif5a2-related eif5a1, which is functional in *eif5a2^K50R/K50R^* mice, is able to substitute for eif5a2 as has been previously reported (Pallmann et al., 2015).

**Fig. 4.**
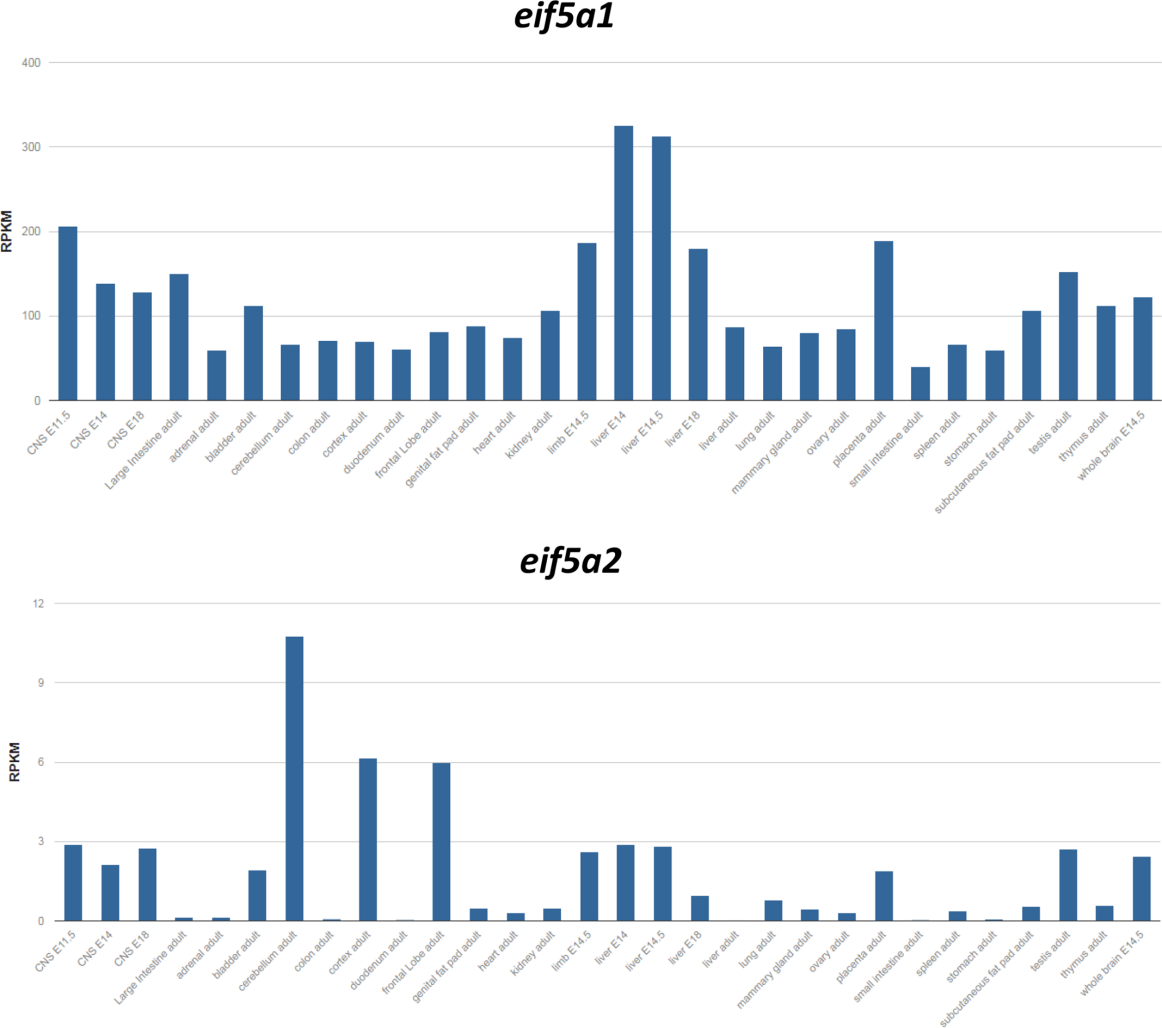
***eif5a1*-*eif5a2* mRNA expression in mouse tissue.** The *eif5a1* and *eif5a2* mRNA tissue expression patterns were analyzed using the mouse ENCODE database that profiles mouse embryonic and adult tissues using RNA sequencing through the R2 Genomics Analysis and Visualization Platform (http://r2.amc.nl). mRNA levels show that *eif5a1* is almost universally expressed in all tissues investigated, whereas *eif5a2* shows a much more restricted expression pattern, at around 30-50 times lower levels. Absolute mRNA levels are expressed as reads per kilobase per million (RKPM)**.**

The apparent embryonic lethality for homozygous *eif5a1*^K50R/K50R^ animals led us to investigate *eif5a1* and *eif5a2* mRNA expression distribution in the mouse using the mouse ENCODE study database that profiles mouse embryonic and adult tissues using RNA sequencing. Raw ENCODE data were uploaded from the NCBI GEO website and analyzed using the R2 Genomics Analysis and Visualization Platform (http://r2.amc.nl). Fig. 4 shows that *eif5A1* is almost universally expressed in all 30 tissues investigated, with expression levels predominantly between ∼50 and ∼200, and up to ∼300 reads per kilobase per million mapped reads (RPKM) in embryonic liver. In contrast, *eif5a2* shows a much more restricted expression pattern, with expression levels of ∼0.1 to ∼3, and up to ∼6-10 RPKM in some brain tissues, or about 30-50 times lower levels than *eif5a1*. These analyses indicate that *eif5a2* could have a distinct function, but only in select tissues, and may not be able to substitute for *eif5a1* in most other tissues.

**Fig. 5.**
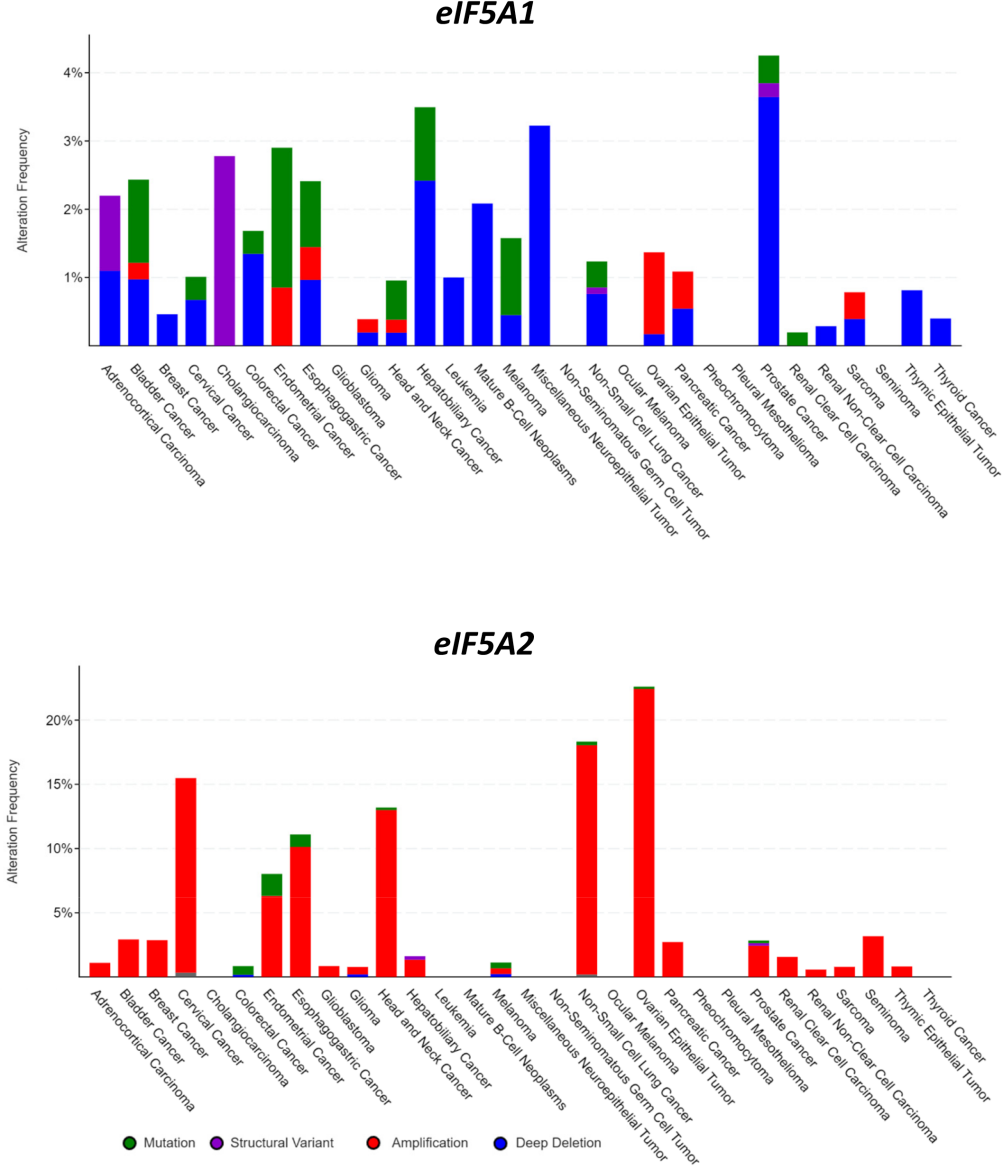
***eIF5A1-eIF5A2* gene aberrations in human cancer.** An overview of *eIF5A1* and *eIF5A2* gene aberrations in human cancer was obtained using the cBioPortal website for Cancer Genomics (https://www.cbioportal.org/). Quantitative analysis of *eIF5A1/2* genetic abnormalities using the 32 TCGA Pan Cancer studies, with 10,967 samples representing most major human cancer types. Analyses was performed using website default settings.

**Fig. 6.**
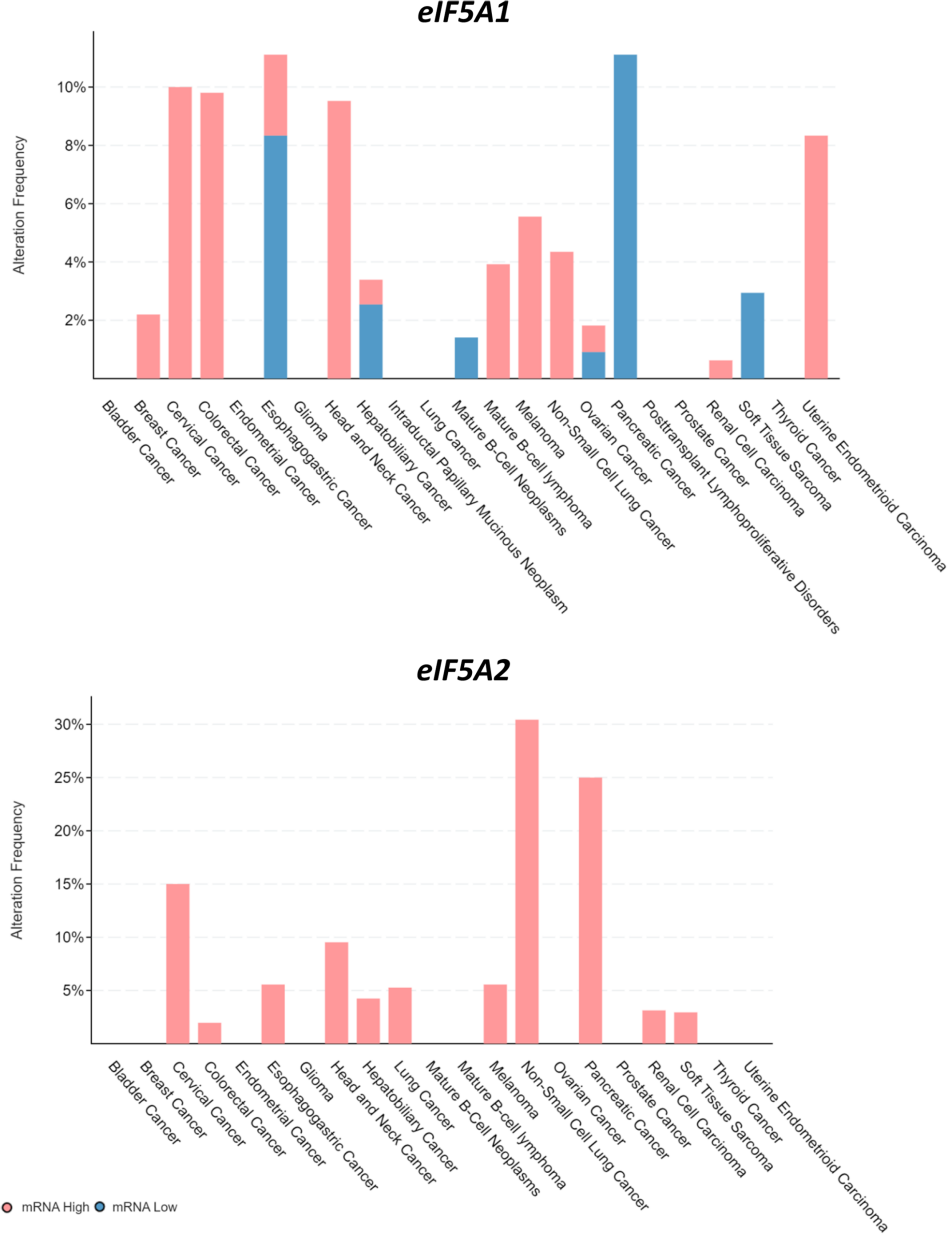
***eIF5A1-eIF5A2* mRNA expression in human cancer.** An overview of *eIF5A1/2* mRNA expression in human cancer, using the ICGC/TCGA dataset (ICGC/TCGA Pan-Cancer Analysis of Whole Genomes Consortium) with 2922 samples representing 33 major human cancer types. Analyses were performed in cBioPortal as in Fig. 5.

The mouse models generated in this study are new tools useful to study human eIF5A1 and eIF5A2 function in neurological disorders, cancer, viral infections, and other diseases. Both genes can have mutations or expression abnormalities in cancer. For an overview, we interrogated the cBioPortal website on human cancer genomic studies. Analysis of the 32 TCGA Pan Cancer studies gave information on *eIF5A1/2* gene aberrations in most major human cancer types (Fig. 5), and of the ICGC/TCGA Pan-Cancer set showed *eIF5A1/2* mRNA expression abnormalities (Fig. 6).

The *eIF5A1* gene was mutated, structurally aberrant, or had gene copy number loss or gain in most major cancer types, but at levels of only up to 4%. In contrast, it showed mRNA over-expression in up to 10% of tumors including cervical, colorectal, head and neck, and uterine cancers. The heterozygous *eif5a1-K50R* (*eif5a1^K50R/+^*) mouse model appears useful to study the effect of partial hypusination loss in these cancers. The *eIF5A2* gene showed a markedly different pattern in cancer, with six tumor types exhibiting more than 5% frequency of eIF5A2 aberrations. The most predominant gene aberration was gene copy number gain, and this could be in up to 22% of tumor samples including cervical, endometrial, esophageal, head and neck, non-small cell lung, and ovarian cancers. Indeed, *eIF5A2* showed mRNA over-expression in most of these cancer types. The homozygous *eif5a2^K50R/K50R^* mice will be an excellent animal model to analyze hypusine-dependent function in these tumors.

**Fig. 7.**
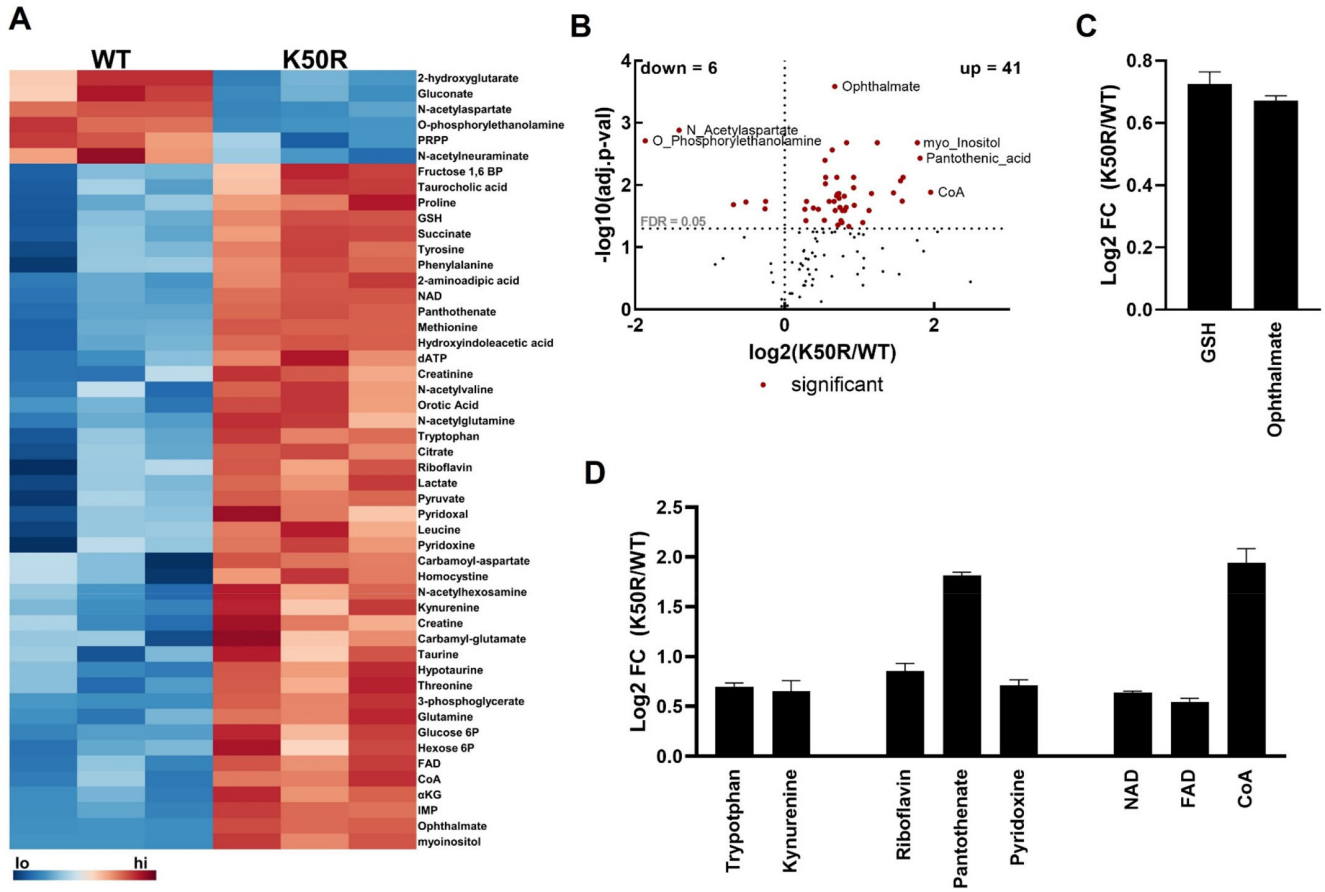
**Metabolomics of primary mouse fibroblasts harboring *eif5a2-K50R* mutation reveal broad metabolic changes.** (A) A heatmap of significantly differentially abundant metabolites between wild-type and *eif5a2^K50R/K50R^*-derived fibroblasts. (B) A volcano plot reveals that 47 metabolites are differentially abundant, with 41 metabolites showing increased levels (FDR<0.05) in K50R cells. Within these significantly elevated metabolites, interesting patterns emerged with glutathione metabolism (C), and vitamins and cofactors (D). Data are expressed as mean±s.e. GSH, glutathione; NAD, nicotinamide adenine dinucleotide; FAD, flavin adenine dinucleotide; CoA, coenzyme A. WT, wild-type.

To gain more insights into the biochemical responses of cells that only express eif5a2 protein without hypusine, we produced a comprehensive metabolomic landscape profile for primary ear skin-derived mouse fibroblasts from wild-type and *eif5a2 ^K50R/K50R^* mice using LC/MS for targeted metabolomics. Of the 126 compounds detected at 5-fold or more over blanks, 53 were significantly (FDR<0.1) differentially abundant between wild-type and *eif5a2^K50R/K50R^* cells with the majority (47/53) elevated in *eif5a2^K50R/K50R^* (Fig. 7A,B). Reduced glutathione (GSH) and ophthalmate, which arise from the same enzymatic pathway, were both elevated in primary mouse dermal fibroblasts prepared from *eif5a2^K50R/K50R^* mice (Fig. 7C). Additionally, we observed increases in (1) tryptophan and kynurenine, which are precursors of *de novo* nicotinamide adenine dinucleotide (NAD) synthesis, (2) the B-vitamins riboflavin (B2), pantothenate (B5), and pyridoxine (B6), and (3) the metabolic cofactors NAD, flavin adenine dinucleotide (FAD), and coenzyme A (CoA) (Fig. 7D). Collectively, these data suggest a general increase in metabolic activity in *eif5a2^K50R/K50R^* cells and may indicate increased biosynthetic rates and higher proliferation in these cells. To further verify this, we compared the proliferation rates of *eif5a2 ^K50R/K50R^* fibroblast cells and wild-type fibroblast cells and found that the K50R-mutated cells grew much more rapidly than wild-type cells (Fig. S4), which is in support of our metabolomic data (Fig. 7).

## DISCUSSION

We report new mouse models for eif5a1 and eif5a2 that each feature a single amino acid exchange, K50R, a mutation that was introduced by CRISPR-Cas9 genome editing. K50 is the only amino acid site that undergoes hypusination, a unique post-translational modification that requires the polyamine spermidine as substrate and the two-step enzymatic reactions involving DHPS and DOHH (Fig. 1). Our new *in vivo eif5a1* and *eif5a2* models are novel because, unlike previously generated gene knockouts, our mice synthesize the full-length proteins with a K50R mutation, which leads to impaired hypusination in homozygous *eif5a2^K50R/K50R^* mice. Previous null-mutant (knockout) mouse models for *eif5a1*, *Dhps*, and *Dohh* revealed that homozygous null-mutant mice were embryonic lethal while heterozygous mice were viable and fertile (Kar et al., 2021; Nishimura et al., 2012; Pallmann et al., 2015; Sievert et al., 2014). In contrast, both heterozygous and homozygous eif5a2 null-mutant mice were viable and fertile without obvious phenotypes, suggesting that the eif5a2 isoform is not essential for embryonic development or steady-state viability in adult mice. It is possible that eIF5A1 functions as replacement for eIF5A2, because the human eIF5A2 protein is 84% identical and 94% similar to human eIF5A1 protein over the entire sequence (Jenkins et al., 2001). However, eIF5A2 is not able to replace the function of eIF5A1, probably because it is expressed only in selected tissues and at lower concentrations.

In a similar manner, we were able to breed viable and fertile heterozygous *eif5a1^K50R/+^* mice but were unable to breed homozygous *eif5a1^K50R/K50R^* mice, suggesting embryonic lethality. As was the case for homozygous *eif5a2* null mice (Pallmann et al., 2015), our homozygous *eif5a2^K50R/K50R^* mice were viable and fertile. The role of eIF5A hypusination in protein synthesis is well defined but since the involvement of these unique proteins is becoming apparent in many additional biological processes and diseases restructure sentence to read better, our K50R mutant mice will be excellent tools for investigators to study the role of hypusination of eIF5A1 and eIF5A2 without the need to genetically knockout the genes.

To better understand the importance and consequence of hypusination on the mouse metabolome, we characterized homozygous *eif5a2^K50R/K50R^* mice that express eif5a2 protein but without the ability to post-translationally synthesize hypusine. While we confirmed in mouse brain homogenates that eif5a2 is not hypusinated, we also noted a decrease in total eif5a2 protein compared to wild-type brain tissues. The reason for this observation is not clear and requires further investigation. We did not identify any whole body-to-organ mass ratio differences or gross abnormalities in multiple organs of *eif5a2^K50R/K50R^* mice compared to wild-type mice (Figs S2 and S3). However, we detected significant metabolic alterations in primary skin fibroblast of mice that produced non-hypusinated eIF5A2 proteins.

While defining metabolic mechanisms is beyond the scope of this work, our data support previous observations linking eIF5A to metabolic phenotypes (Li et al., 2022; Puleston et al., 2019; Zhou et al., 2022) and reveal interesting patterns that may help direct future investigations. First, GSH and ophthalmate, which were elevated in *eif5a2^K50R/K50R^* fibroblasts, are formed from the substrates glutamine or γ-aminobutyrate, respectively, by the serial action of γ-glutamylcysteine synthetase (GCS) and glutathione synthetase (GS) (Orlowski and Wilk, 1978). GSH feedback inhibits GCS (Richman and Meister, 1975) and, when oxidized to GSSG by oxidative stress, the inhibition is removed allowing for both GSH and ophthalmate synthesis to proceed. Since in oxidative conditions *de novo* synthesized GSH will proceed to GSSG but ophthalmate will accumulate, ophthalmate has been suggested to be a marker of oxidative stress (Soga et al., 2006). In support of this, we observed elevation of TCA cycle intermediates (citrate, alpha-ketoglutarate, succinate) and required cofactors (NAD, CoA). Since mitochondrial electron transport chain activity is a principal source of oxidant production, our data suggest increased glutathione synthesis may be a compensatory response to enhanced mitochondrial activity in *eif5a2^K50R/K50R^* cells.

Secondly, we observed increased abundances of B-vitamins (riboflavin, pantothenate, and pyridoxine) and cofactors and their biosynthetic intermediates including tryptophan and kynurenine, which give rise to NAD, pantothenate, which is the precursor for CoA biosynthesis, and FAD. Together these vitamins and cofactors are involved in a host of metabolic and biosynthetic processes. Though the mechanism is undefined, it is clear that *eif5a2^K50R/K50R^* cells have a strong metabolic phenotype that should be explored further.

In addition to FABAS, other neurodevelopmental disorders have been linked to genes that regulate the polyamine pathway (Fig. 1). Gain-of-function mutations in the C-terminus of ODC have been connected to Bachmann-Bupp Syndrome (BABS; OMIM #619075) (Bupp et al., 2018; Rajasekaran et al., 2021; VanSickle et al., 2021; Bupp et al., 2022) and loss-of-function mutations in spermine synthase are associated with Snyder-Robinson Syndrome (SRS) (Albert et al., 1993; Cason et al., 2003; Starks et al., 2018). Patients with gene mutations in DHPS and bi-allelic variants in DOHH have recently been identified (Ganapathi et al., 2019; Park et al., 2022; Ziegler et al., 2022). The DHPS-related disorder is referred to as DHPS deficiency or DHPS syndrome (https://rarediseases.org/rare-diseases/deoxyhypusine-synthase-disorder/) and is also known as neurodevelopmental disorder with seizures and speech and walking impairment (NEDSSWI; OMIM #618480). The DOHH-related disorder is referred to as neurodevelopmental disorder with microcephaly, cerebral atrophy, and visual impairment (NEDMVIC; OMIM #620066). The three proteins eIF5A1, DHPS, and DOHH are highly conserved in eukaryotes and their pathogenic variants are collectively referred to as eIF5A1 and hypusination-related disorders that are characterized by global developmental delay, intellectual disability, facial dysmorphism, and microcephaly (Ziegler et al., 2022). It is likely that other polyamine-regulating enzymes such as SAMDC, MTA, SSAT, PAO, and SMOX as well as polyamine uptake transporters (e.g. ATP13A2-5) are also affiliated with neurodevelopmental disorders as all of them co-regulate the critical balance of polyamines in the human body. Substantial dysregulation of putrescine, spermidine or spermine levels can cause unbalanced polyamine ratios leading to unfavorable events during embryonic and infant development. Moreover, spermidine-induced hypusination preserves mitochondrial function and improves cognitive function during aging (Hofer et al., 2021; Schroeder et al., 2021).

In addition to providing new tools for the study of the physiological role of hypusination *in vivo*, our hypusination-impaired (eif5a1) or -deficient (eif5a2) mouse models will be very useful for future investigations in developing therapies against hypusination-related disorders caused by variants of eIF5A1, DHPS and DOHH. In a similar manner, the anti-protozoan ODC inhibitor difluoromethylornithine (DFMO) (Fig. 1) was effective in an ODC mouse model (Soler et al., 1996) and we recently repositioned DFMO for the treatment of a BABS patient, with remarkable disease improvements (Rajasekaran et al., 2021). Moreover, elevated eIF5A2 expression carries unfavorable prognostic implications for several cancer and both eIF5A1 and eIF5A2 have been advanced as cancer biomarkers (Bai et al., 2018; Caraglia et al., 2013; Clement et al., 2006; Liu et al., 2016; Mathews and Hershey, 2015; Nakanishi and Cleveland, 2016; Pallmann et al., 2015; Wang et al., 2013), thus further expanding the potential use for our new mouse models. Finally, eIF5A2 regulates the expression of antiviral genes (Farache et al., 2022) and our *eif5a2^K50R/K50R^* mouse model could serve as a novel *in vivo* system to study antiviral drugs.

These new mouse lines represent excellent *in vivo* models to study hypusine-dependent biological processes, hypusination-related disorders caused by eIF5A1 and eIF5A2 gene aberrations and mRNA expression dysregulation as well as several major human cancer types and potential therapies.

## MATERIALS AND METHODS

This study was conducted according to a protocol that was approved by the Institutional Animal Care and Use Committee at Michigan State University. Both male and female mice were used in analyses.

### Generation of *eif5a1^K50R/+^* mice

CRISPR-Cas9 genome editing was used to generate *eif5a1-K50R* (AAG>CGG) mutant mice. A guide RNA (gRNA) with a protospacer and PAM (5′ N_20_-*NGG*-3′) sequences 5′- ACTTCGAAGACTGGCAAGCA *TGG* −3′, was used to target exon 3 of the mouse *eif5a1* gene (ENSMUSG00000078812). A single-stranded oligodeoxynucleotide (ssODN) with sequence 5′-GTGGGAGCTGCAGGGAAGGCAAGATGTTGAGAGGTGATTCTAACCTTGGCATGGCCATGCCGGCCAGTCTTCGAAGTAGACATCTCGACGATCTTAC-3′ was used as a donor template carrying the K50R mutation. Recombinant S.p. Cas9 protein, synthetic tracrRNA, and crRNA (Integrated DNA Technologies) were used in a ribonucleoprotein (RNP) format for CRISPR-Cas9 editing. Briefly, tracrRNA and crRNA were incubated at 95°C for 5 min and allowed to cool down to form RNA hybrids, which were then incubated with Cas9 protein for 5 min at 37°C to pre-form RNP complexes. RNPs with ssODN were introduced into 129X1/svJ zygotes by electroporation as described previously (RNP at 50 ng/µl; ssODN at 0.75 µM; 30 V for two pulses) (Qin et al., 2015). Embryos were allowed to recover and implanted into pseudo-pregnant foster females following standard procedures. Mutant analysis of offspring was performed using PCR, a T7 Endonuclease I assay (New England BioLabs, #M0302L) and Sanger sequencing. The heterozygous *eif5a1^K50R/+^* mouse model was generated and maintained on a 129X1/SvJ genomic background (129X1/SvJ-*Eif5a^em1Bach^*/Mmmh; RRID:MMRRC_068486-MU).

### Generation of *eif5a2^K50R/K50R^* mice

CRISPR-Cas9 genome editing, as described above for eif5a1, was used to generate *eif5a2-K50R* (AAG>CGA) mutant mice as well as a K50-G52 deleted allele. A gRNA with a protospacer and PAM sequences 5′-ACTTCCAAGACTGGGAAGCA *TGG* −3′, was used to target exon 2 of the mouse eif5a2 gene (ENSMUSG00000050192). An ssODN with sequence 5′ATGATCTTAACGGTGAAGCCAAAAAATGACTTGGAAGGGCCTACCTTGGCATGACCATGTCGCCCAGTCTTGGAAGTTGACATCTCCACGATTT −3′ was used as a repair template carrying the K50R mutation. RNPs with ssODN were electroporated either into C57Bl/6 or 129×1/svJ zygotes as described above. The homozygous *eif5a2^K50R/K50R^* mouse model was first generated on C57BL/6J and 129X1/SvJ genomic backgrounds but was subsequently backcrossed onto a CD1 background to improve litter size (Crl:CD1-*Eif5a2^em1Bach^*/Mmmh; RRID:MMRRC_068487-MU).

### Genotyping

For genotyping, tail biopsies were lysed with proteinase K to release genomic DNA for PCR analysis. Primers used for genotyping are listed below: pair O203/O206 for a product of 327 bp O203_eif5a1_genF1: CCATCGTTACTGACCCTAGTTC and O206_eif5a1_genR2: GAAAACAGCAAAAGGGGTCAAGA. Pair O208/O209 for a product of 503 bp O208_eif5a2_genF2: AGTTCCCTCAGAAAAACCGCA and O209_eif5a2_genR1: ACCACACACGTCAGCAAGTC, or pair O319/O320 for smaller products: 135 bp O319_eif5a2-F3: TCAAAGGGCGACCATGCAAA and O320 eif5a2-R3: TCACGATGTAGAATGGAACACG. Pair O343/O344 for big deletion products: 1034 bp O343_eif5a2_genF4: GTCCCAGTCTTCCCCGAAC and O344_eif5a2_genR4: TACACAGCAGCACAATCCCA. The new heterozygous *eif5a1^K50R/+^* and homozygous *eif5a2^K50R/K50R^* mouse models as well as a related homozygous *eif5a2^ΔK50-G52/ΔK50-G52^* deletion model on a 129X1/SvJ background (a 9-bp deletion which results in the removal of three amino acids K50-G52) are available from the Mutant Mouse Resource and Research Centers (MMRRC) at the University of Missouri (Columbia, MO, USA), supported by the National Institutes of Health (NIH). A list of the newly generated *eif5a1* and *eif5a2* mouse models is shown in Table 1, also including the MMRRC identifier numbers. The O208/O209 primer pair resulting in a 503 bp product was used for *eif5a2-K50R* genotyping in this manuscript. The resulting PCR product was digested using *Pfl*FI. The *eif5a2-K50R* mutant PCR product is cut into two bands, while the wild-type product is not digested. (Fig. 3A).

### Western blotting

Mouse brain tissue was prepared in IP Lysis buffer provided in the Pierce™ Direct Magnetic IP/Co-IP Kit (ThermoFisher Scientific). Total protein concentration was determined using the Bradford dye reagent protein assay (Bio-Rad Laboratories). Cell lysates in SDS sample buffer were boiled for 10 min and equal amounts of protein were resolved by 12% SDS-PAGE. Protein was electro-transferred onto 0.45 µM polyvinylidene difluoride Immobilon-P membrane (Millipore). Primary antibodies, rabbit anti-Hypusine (ABS1064, Millipore), mouse anti-eIF5A2 (TA505100, Origene) and mouse anti-β-actin (SC-47778, Santa Cruz Biotechnology) were incubated overnight at 4°C in 5% BSA in Tris-buffered saline containing 0.1% Tween-20. The anti-hypusine antibody identifies the hypusine modification as well as the deoxyhypusine intermediate. The mouse anti-eIF5A2 antibody (TA505100, Origene) was tested to be specific for eIF5A2 protein only and did not detect eIF5A1 protein by western blot when using commercially available HEK293 cell lysates that overexpress either eIF5A1 or eIF5A2 (LC419616 or LC412495, Origene) (Fig. S5). Secondary antibodies were incubated for 1 h at room temperature in 5% non-fat dry milk in Tris-buffered saline containing 0.1% Tween-20. Blots were imaged using an Odyssey Clx (Licor) western blot scanner.

### Mouse organ weight measurements

Wild-type and *eif5a2^K50R/K50R^* male and female mice were weighed prior to euthanasia. The brain, lungs, liver, spleen, kidney and testis were removed and weighed. Organ weights were normalized to total mouse weight and represented as percent of total mouse body weight.

### Isolation of primary mouse dermal fibroblasts

Primary mouse dermal fibroblasts were isolated from ear punch samples derived from wild-type and *eif5a2^K50R/K50R^* mutant CD1 mice. Briefly, the ears of the mice were sterilized using 70% ethanol prior to extracting 2 mm round punch biopsies from one mouse of each genotype. The ear punch biopsies were placed into separate wells of a six-well plate. The pieces were covered with a 20 mm round coverslip to keep it attached to the plate. A few drops of complete growth medium were carefully added under each coverslip. Growth medium was gently added to the rest of the well. The outgrowth of cells was monitored daily. Medium was changed every 3-4 days. Once the wells became confluent, the cells were detached using 0.25% trypsin/2.21 mM EDTA (Corning) and passed into larger dishes for use in experiments. All cells were maintained in DMEM medium (Gibco) supplemented with 10% heat-inactivated fetal bovine serum (Hyclone), penicillin (100 IU/ml), and streptomycin (100 µg/ml), non-essential amino acids (0.1 mM) and sodium pyruvate (1 mM). All cell cultures are routinely monitored for mycoplasma contamination every 6 months using the MycoAlert™ PLUS Mycoplasma Detection Kit (Lonza).

### Microscopy

Light micrographs of primary mouse dermal fibroblasts were captured at 10X using a Leica DMi1 microscope equipped with a camera.

### Cell growth assay

The colorimetric SRB assay was used to measure primary mouse dermal fibroblast cell growth from 0 to 72 h. Briefly, dermal fibroblasts were plated in transparent flat 96-well plates and allowed to attach overnight (time zero). Cells were then fixed at time zero, 24, 48 and 72 h with 10% TCA at 4°C for 1 h, washed with deionized water, and dried at room temperature. Cells were stained with 100 µl of 0.4% SRB in 1% acetic acid for 20 min at room temperature, rinsed five times with 1% acetic acid and allowed to dry at room temperature. One hundred µl of 10 mM Tris-HCl pH 7.0 was added to each well, shaken for 10 min at room temperature and read at 540 nm using a Spectramax Flexstation microplate reader. Increased absorbance at 540 nm correlated to increased cell growth.

### Cell proliferation assay

Cell proliferation of the primary mouse dermal fibroblasts was determined by measuring BrdU incorporation in DNA synthesis using the BrdU Cell Proliferation Assay (Millipore) according to the manufacturer's protocol. Briefly, cells were plated in transparent flat 96-well plates and allowed to attach overnight. BrdU was then added and allowed to incorporate for 24 h. The cells were then fixed, washed, and incubated with the provided anti-BrdU monoclonal antibody for 1 h at room temperature. The cells were again washed and then incubated with the provided goat anti-mouse IgG peroxidase conjugate for 30 min at room temperature. After washing, the cells were incubated with TMB peroxidase substrate for 30 min at room temperature in the dark. Stop solution was then added to the cells and the absorbance at 450 nm was measured using a Spectramax Flexstation microplate reader. Cells that did not receive BrdU labeling were used as a background control.

### Pathology

Six *eif5a2^K50R/K50R^* mice (three males and three females) and two wild-type mice (one male and one female) were euthanized by CO_2_ inhalation followed by cervical dislocation. Gross dissection, examination, and sampling of all organs included spleen, lung, liver, heart, adrenal gland, thymus, ovary, testicle, uterus, kidney, and brain. Tissues were fixed in 10% neutral buffered formalin for 48 h, then transferred to 70% ethanol before being processed into paraffin for histologic examination. Five-micron-thick sections of paraffin embedded blocks were fixed to charged slides and stained with Hematoxylin and Eosin and examined by light microscopy by a board-certified veterinary pathologist (DWA). Representative images of selected organs are shown in Fig. S3.

### Bio-informatic analyses

The *eif5a1* and *eif5a2* mRNA expression patterns in mouse tissues (Fig. 4) were generated using the mouse ENCODE database (Yue et al., 2014). ENCODE raw data were retrieved from the public Gene Expression Omnibus (GEO) dataset on the NCBI website (http://www.ncbi.nlm.nih.gov/geo/) under number GSE49847 and analyzed using the R2 Genomics Analysis and Visualization Platform developed in the Department of Oncogenomics at the University of Amsterdam Academic Medical Center (http://r2.amc.nl). Absolute mRNA levels are expressed in RPKM (Reads Per Kilobase Million). The eIF5A1 and eIF5A2 gene aberrations and mRNA expression in human cancers were analyzed using the cBioPortal website for Cancer Genomics (https://www.cbioportal.org/). For the overview of frequent *eIF5A1/2* genetic abnormalities (Fig. 5), the 32 TCGA Pan Cancer studies were used, with 10,967 samples representing most major human cancer types. For a first overview of *eIF5A1/2* mRNA expression in human cancer (Fig. 6), the ICGC/TCGA dataset (ICGC/TCGA Pan-Cancer Analysis of Whole Genomes Consortium) was used, with 2922 samples representing 33 major human cancer types. Analyses were performed using website default settings.

### LC/MS metabolomics

Cell media was aspirated, cells were washed rapidly in 0.9% NaCl and plates snap-frozen on dry ice. Metabolites were extracted from 1×10^6^ cells by modified Bligh-Dyer extraction (Bligh and Dyer, 1959). Briefly, ice cold methanol was added directly to frozen cells. Plates were then scraped, and the methanol-cell slurry transferred to a 1.5 mL Eppendorf tube to which one volume of chloroform was added. One part chloroform was added to the extract, vortexed for 10 s, and incubated on ice for 30 min. 0.9 parts water was added and vigorously vortexed to achieve phase separation. Samples were centrifuged to phase separate the extracts, the top layer containing polar metabolites was aliquoted into a fresh tube and dried in a speed vac. Samples were then resuspended in H_2_O for LC/MS analysis. Metabolite profiling analysis was completed using ion-paired reversed phase liquid chromatography using an HPLC (1290 Infinity II, Agilent Technologies, Santa Clara, CA, USA) coupled to a triple quadruple mass spectrometer (6470, Agilent Technologies) with electrospray ionization operated in negative mode as described previously (Roy et al., 2020). Data were analyzed in MassHunter Quantitative Analysis software (v9.0, Agilent Technologies) and peaks visually reviewed by a trained technician blinded to experimental conditions.

### Statistical analyses

For metabolomics, differential abundance of metabolites was completed using Metaboanalyst (v 5.0, PMID: 35715522) using a false discovery rate (FDR) of 0.1. All data are expressed as means±s.e. The statistical significance for experiments comparing wild-type and eif5a2-K50R mouse brain lysates and dermal fibroblasts was determined using an unpaired Student's *t*-test assuming the null hypothesis.

## Supplementary Material

10.1242/biolopen.059647_sup1 Supplementary information

## References

[BIO059647C1] Albert, J., Schwartz, C. E., Boerkoel, C. F. and Stevenson, R. E. (1993). Snyder-robinson syndrome. In *GeneReviews*^®^ [Internet] (ed. M. P. Adam, H. H. Ardinger, R. A. Pagon, S. E. Wallace, L. J. H. Bean, K. Stephens and A. Amemiya). Seattle, WA: University of Washington. 23805436

[BIO059647C2] Bai, H.-Y., Liao, Y.-J., Cai, M.-Y., Ma, N.-F., Zhang, Q., Chen, J.-W., Zhang, J.-X., Wang, F.-W., Wang, C.-Y., Chen, W. H. et al. (2018). Eukaryotic initiation factor 5A2 contributes to the maintenance of CD133(+) hepatocellular carcinoma cells via the c-Myc/microRNA-29b Axis. *Stem Cells* 36, 180-191. 10.1002/stem.2734 29119708

[BIO059647C3] Bandino, A., Geerts, D., Koster, J. and Bachmann, A. S. (2014). Deoxyhypusine synthase (DHPS) inhibitor GC7 induces p21/Rb-mediated inhibition of tumor cell growth and DHPS expression correlates with poor prognosis in neuroblastoma patients. *Cell. Oncol.* 37, 387-398. 10.1007/s13402-014-0201-9 PMC13004463 25315710

[BIO059647C4] Bligh, E. G. and Dyer, W. J. (1959). A rapid method of total lipid extraction and purification. *Can. J. Biochem. Physiol.* 37, 911-917. 10.1139/y59-099 13671378

[BIO059647C5] Bupp, C. P., Schultz, C. R., Uhl, K. L., Rajasekaran, S. and Bachmann, A. S. (2018). Novel de novo pathogenic variant in the ODC1 gene in a girl with developmental delay, alopecia, and dysmorphic features. *Am. J. Med. Genet. A* 176, 2548-2553. 10.1002/ajmg.a.40523 30239107

[BIO059647C500] Bupp, C, Michael, J, VanSickle, E, Rajasekaran, S and Bachmann, A. S. (2022). Bachmann-Bupp syndrome. In *GeneReviews*^®^ [Internet] (ed. M. P. Adam, D. B. Everman, G. M. Mirzaa, R. A. Pagon, S. E. Wallace, L. J. H. Bean, K. W. Gripp and A. Amemiya). Seattle, WA: University of Washington. 36007106

[BIO059647C6] Caraglia, M., Park, M. H., Wolff, E. C., Marra, M. and Abbruzzese, A. (2013). eIF5A isoforms and cancer: two brothers for two functions? *Amino Acids* 44, 103-109. 10.1007/s00726-011-1182-x 22139412 PMC3536922

[BIO059647C7] Cason, A. L., Ikeguchi, Y., Skinner, C., Wood, T. C., Holden, K. R., Lubs, H. A., Martinez, F., Simensen, R. J., Stevenson, R. E., Pegg, A. E. et al. (2003). X-linked spermine synthase gene (SMS) defect: the first polyamine deficiency syndrome. *Eur. J. Hum. Genet.* 11, 937-944. 10.1038/sj.ejhg.5201072 14508504

[BIO059647C8] Clement, P. M., Johansson, H. E., Wolff, E. C. and Park, M. H. (2006). Differential expression of eIF5A-1 and eIF5A-2 in human cancer cells. *FEBS J.* 273, 1102-1114. 10.1111/j.1742-4658.2006.05135.x 16519677 PMC4406228

[BIO059647C9] Coni, S., Serrao, S. M., Yurtsever, Z. N., Di Magno, L., Bordone, R., Bertani, C., Licursi, V., Ianniello, Z., Infante, P., Moretti, M. et al. (2020). Blockade of EIF5A hypusination limits colorectal cancer growth by inhibiting MYC elongation. *Cell Death Dis.* 11, 1045. 10.1038/s41419-020-03174-6 33303756 PMC7729396

[BIO059647C10] Farache, D., Liu, L. and Lee, A. S. Y. (2022). Eukaryotic initiation factor 5A2 regulates expression of antiviral genes. *J. Mol. Biol.* 434, 167564. 10.1016/j.jmb.2022.167564 35358571 PMC11906106

[BIO059647C11] Faundes, V., Jennings, M. D., Crilly, S., Legraie, S., Withers, S. E., Cuvertino, S., Davies, S. J., Douglas, A. G. L., Fry, A. E., Harrison, V. et al. (2021). Impaired eIF5A function causes a Mendelian disorder that is partially rescued in model systems by spermidine. *Nat. Commun.* 12, 833. 10.1038/s41467-021-21053-2 33547280 PMC7864902

[BIO059647C12] Ganapathi, M., Padgett, L. R., Yamada, K., Devinsky, O., Willaert, R., Person, R., Au, P. B., Tagoe, J., Mcdonald, M., Karlowicz, D. et al. (2019). Recessive rare variants in deoxyhypusine synthase, an enzyme involved in the synthesis of hypusine, are associated with a neurodevelopmental disorder. *Am. J. Hum. Genet.* 104, 287-298. 10.1016/j.ajhg.2018.12.017 30661771 PMC6369575

[BIO059647C13] Gutierrez, E., Shin, B. S., Woolstenhulme, C. J., Kim, J. R., Saini, P., Buskirk, A. R. and Dever, T. E. (2013). eIF5A promotes translation of polyproline motifs. *Mol. Cell* 51, 35-45. 10.1016/j.molcel.2013.04.021 23727016 PMC3744875

[BIO059647C14] Hofer, S. J., Liang, Y., Zimmermann, A., Schroeder, S., Dengjel, J., Kroemer, G., Eisenberg, T., Sigrist, S. J. and Madeo, F. (2021). Spermidine-induced hypusination preserves mitochondrial and cognitive function during aging. *Autophagy* 17, 2037-2039. 10.1080/15548627.2021.1933299 34105442 PMC8386697

[BIO059647C15] Jenkins, Z. A., Haag, P. G. and Johansson, H. E. (2001). Human eIF5A2 on chromosome 3q25-q27 is a phylogenetically conserved vertebrate variant of eukaryotic translation initiation factor 5A with tissue-specific expression. *Genomics* 71, 101-109. 10.1006/geno.2000.6418 11161802

[BIO059647C16] Kar, R. K., Hanner, A. S., Starost, M. F., Springer, D., Mastracci, T. L., Mirmira, R. G. and Park, M. H. (2021). Neuron-specific ablation of eIF5A or deoxyhypusine synthase leads to impairments in growth, viability, neurodevelopment, and cognitive functions in mice. *J. Biol. Chem.* 297, 101333. 10.1016/j.jbc.2021.101333 34688659 PMC8605248

[BIO059647C17] Kim, H. I., Schultz, C. R., Chandramouli, G. V. R., Geerts, D., Risinger, J. I. and Bachmann, A. S. (2022). Pharmacological targeting of polyamine and hypusine biosynthesis reduces tumor activity of endometrial cancer. *J. Drug Target* 30, 623-633. 10.1080/1061186X.2022.2036164 35100927

[BIO059647C18] Landau, G., Bercovich, Z., Park, M. H. and Kahana, C. (2010). The role of polyamines in supporting growth of mammalian cells is mediated through their requirement for translation initiation and elongation. *J. Biol. Chem.* 285, 12474-12481. 10.1074/jbc.M110.106419 20181941 PMC2857121

[BIO059647C19] Li, H., Wu, B. K., Kanchwala, M., Cai, J., Wang, L., Xing, C., Zheng, Y. and Pan, D. (2022). YAP/TAZ drives cell proliferation and tumour growth via a polyamine-eIF5A hypusination-LSD1 axis. *Nat. Cell Biol.* 24, 373-383. 10.1038/s41556-022-00848-5 35177822 PMC8930503

[BIO059647C20] Liu, R. R., Lv, Y. S., Tang, Y. X., Wang, Y. F., Chen, X. L., Zheng, X. X., Xie, S. Z., Cai, Y., Yu, J. and Zhang, X. N. (2016). Eukaryotic translation initiation factor 5A2 regulates the migration and invasion of hepatocellular carcinoma cells via pathways involving reactive oxygen species. *Oncotarget* 7, 24348-24360. 10.18632/oncotarget.8324 27028999 PMC5029706

[BIO059647C21] Mastracci, T. L., Robertson, M. A., Mirmira, R. G. and Anderson, R. M. (2015). Polyamine biosynthesis is critical for growth and differentiation of the pancreas. *Sci. Rep.* 5, 13269. 10.1038/srep13269 26299433 PMC4547391

[BIO059647C22] Mathews, M. B. and Hershey, J. W. (2015). The translation factor eIF5A and human cancer. *Biochim. Biophys. Acta* 1849, 836-844. 10.1016/j.bbagrm.2015.05.002 25979826 PMC4732523

[BIO059647C23] Nakanishi, S. and Cleveland, J. L. (2016). Targeting the polyamine-hypusine circuit for the prevention and treatment of cancer. *Amino Acids* 48, 2353-2362. 10.1007/s00726-016-2275-3 27357307 PMC5573165

[BIO059647C24] Nishimura, K., Lee, S. B., Park, J. H. and Park, M. H. (2012). Essential role of eIF5A-1 and deoxyhypusine synthase in mouse embryonic development. *Amino Acids* 42, 703-710. 10.1007/s00726-011-0986-z 21850436 PMC3220921

[BIO059647C25] Olsen, M. E., Cressey, T. N., Muhlberger, E. and Connor, J. H. (2018). Differential mechanisms for the involvement of polyamines and hypusinated eIF5A in Ebola virus gene expression. *J. Virol.* 92, e01260-18. 10.1128/JVI.01260-18 30045993 PMC6158423

[BIO059647C26] Orlowski, M. and Wilk, S. (1978). Synthesis of ophthalmic acid in liver and kidney in vivo. *Biochem. J.* 170, 415-419. 10.1042/bj1700415 637852 PMC1183909

[BIO059647C27] Pallmann, N., Braig, M., Sievert, H., Preukschas, M., Hermans-Borgmeyer, I., Schweizer, M., Nagel, C. H., Neumann, M., Wild, P., Haralambieva, E. et al. (2015). Biological relevance and therapeutic potential of the hypusine modification system. *J. Biol. Chem.* 290, 18343-18360. 10.1074/jbc.M115.664490 26037925 PMC4513096

[BIO059647C28] Park, M. H. (2006). The post-translational synthesis of a polyamine-derived amino acid, hypusine, in the eukaryotic translation initiation factor 5A (eIF5A). *J. Biochem.* 139, 161-169. 10.1093/jb/mvj034 16452303 PMC2494880

[BIO059647C29] Park, M. H. and Wolff, E. C. (2018). Hypusine, a polyamine-derived amino acid critical for eukaryotic translation. *J. Biol. Chem.* 293, 18710-18718. 10.1074/jbc.TM118.003341 30257869 PMC6290153

[BIO059647C30] Park, M. H., Cooper, H. L. and Folk, J. E. (1981). Identification of hypusine, an unusual amino acid, in a protein from human lymphocytes and of spermidine as its biosynthetic precursor. *Proc. Natl. Acad. Sci. USA* 78, 2869-2873. 10.1073/pnas.78.5.2869 6789324 PMC319460

[BIO059647C31] Park, J. H., Aravind, L., Wolff, E. C., Kaevel, J., Kim, Y. S. and Park, M. H. (2006). Molecular cloning, expression, and structural prediction of deoxyhypusine hydroxylase: a HEAT-repeat-containing metalloenzyme. *Proc. Natl. Acad. Sci. USA* 103, 51-56. 10.1073/pnas.0509348102 16371467 PMC1324997

[BIO059647C32] Park, M. H., Nishimura, K., Zanelli, C. F. and Valentini, S. R. (2010). Functional significance of eIF5A and its hypusine modification in eukaryotes. *Amino Acids* 38, 491-500. 10.1007/s00726-009-0408-7 19997760 PMC2829442

[BIO059647C33] Park, M. H., Kar, R. K., Banka, S., Ziegler, A. and Chung, W. K. (2022). Post-translational formation of hypusine in eIF5A: implications in human neurodevelopment. *Amino Acids* 54, 485-499. 10.1007/s00726-021-03023-6 34273022 PMC9117371

[BIO059647C34] Pelechano, V. and Alepuz, P. (2017). eIF5A facilitates translation termination globally and promotes the elongation of many non polyproline-specific tripeptide sequences. *Nucleic Acids Res.* 45, 7326-7338. 10.1093/nar/gkx47 28549188 PMC5499558

[BIO059647C35] Puleston, D. J., Buck, M. D., Klein Geltink, R. I., Kyle, R. L., Caputa, G., O'sullivan, D., Cameron, A. M., Castoldi, A., Musa, Y., Kabat, A. M. et al. (2019). Polyamines and eIF5A hypusination modulate mitochondrial respiration and macrophage activation. *Cell Metab.* 30, 352-363.e8. 10.1016/j.cmet.2019.05.003 31130465 PMC6688828

[BIO059647C36] Qin, W., Dion, S. L., Kutny, P. M., Zhang, Y., Cheng, A. W., Jillette, N. L., Malhotra, A., Geurts, A. M., Chen, Y. G. and Wang, H. (2015). Efficient CRISPR/Cas9-mediated genome editing in mice by zygote electroporation of nuclease. *Genetics* 200, 423-430. 10.1534/genetics.115.176594 25819794 PMC4492369

[BIO059647C37] Rajasekaran, S., Bupp, C. P., Leimanis-Laurens, M., Shukla, A., Russell, C., Junewick, J., Gleason, E., Vansickle, E. A., Edgerly, Y., Wittmann, B. M. et al. (2021). Repurposing eflornithine to treat a patient with a rare ODC1 gain-of-function variant disease. *Elife* 10, e67097. 10.7554/eLife.67097 34282722 PMC8291972

[BIO059647C38] Richman, P. G. and Meister, A. (1975). Regulation of gamma-glutamyl-cysteine synthetase by nonallosteric feedback inhibition by glutathione. *J. Biol. Chem.* 250, 1422-1426. 10.1016/S0021-9258(19)41830-9 1112810

[BIO059647C39] Roy, D. G., Chen, J., Mamane, V., Ma, E. H., Muhire, B. M., Sheldon, R. D., Shorstova, T., Koning, R., Johnson, R. M., Esaulova, E. et al. (2020). Methionine metabolism shapes T helper cell responses through regulation of epigenetic reprogramming. *Cell Metab.* 31, 250-266 e9. 10.1016/j.cmet.2020.01.006 32023446

[BIO059647C40] Saini, P., Eyler, D. E., Green, R. and Dever, T. E. (2009). Hypusine-containing protein eIF5A promotes translation elongation. *Nature* 459, 118-121. 10.1038/nature08034 19424157 PMC3140696

[BIO059647C41] Schroeder, S., Hofer, S. J., Zimmermann, A., Pechlaner, R., Dammbrueck, C., Pendl, T., Marcello, G. M., Pogatschnigg, V., Bergmann, M., Muller, M. et al. (2021). Dietary spermidine improves cognitive function. *Cell Rep.* 35, 108985. 10.1016/j.celrep.2021.108985 33852843

[BIO059647C42] Schuller, A. P., Wu, C. C., Dever, T. E., Buskirk, A. R. and Green, R. (2017). eIF5A functions globally in translation elongation and termination. *Mol. Cell* 66, 194-205.e5. 10.1016/j.molcel.2017.03.003 28392174 PMC5414311

[BIO059647C43] Schultz, C. R., Geerts, D., Mooney, M., El-Khawaja, R., Koster, J. and Bachmann, A. S. (2018). Synergistic drug combination GC7/DFMO suppresses hypusine/spermidine-dependent eIF5A activation and induces apoptotic cell death in neuroblastoma. *Biochem. J.* 475, 531-545. 10.1042/BCJ20170597 29295892

[BIO059647C44] Sievert, H., Pallmann, N., Miller, K. K., Hermans-Borgmeyer, I., Venz, S., Sendoel, A., Preukschas, M., Schweizer, M., Boettcher, S., Janiesch, P. C. et al. (2014). A novel mouse model for inhibition of DOHH-mediated hypusine modification reveals a crucial function in embryonic development, proliferation and oncogenic transformation. *Dis. Model. Mech.* 7, 963-976. 10.1242/dmm.014449 24832488 PMC4107325

[BIO059647C45] Singh, K., Martinez, M. G., Lin, J., Gregory, J., Nguyen, T. U., Abdelaal, R., Kang, K., Brennand, K., Grunweller, A., Ouyang, Z. et al. (2022). Transcriptional and translational dynamics of Zika and dengue virus infection. *Viruses* 14, 1418. 10.3390/v14071418 35891396 PMC9316442

[BIO059647C46] Smeltzer, S., Quadri, Z., Miller, A., Zamudio, F., Hunter, J., Stewart, N. J. F., Saji, S., Lee, D. C., Chaput, D. and Selenica, M. B. (2021). Hypusination of Eif5a regulates cytoplasmic TDP-43 aggregation and accumulation in a stress-induced cellular model. *Biochim. Biophys. Acta Mol. Basis Dis.* 1867, 165939. 10.1016/j.bbadis.2020.165939 32882370

[BIO059647C47] Soga, T., Baran, R., Suematsu, M., Ueno, Y., Ikeda, S., Sakurakawa, T., Kakazu, Y., Ishikawa, T., Robert, M., Nishioka, T. et al. (2006). Differential metabolomics reveals ophthalmic acid as an oxidative stress biomarker indicating hepatic glutathione consumption. *J. Biol. Chem.* 281, 16768-16776. 10.1074/jbc.M601876200 16608839

[BIO059647C48] Soler, A. P., Gilliard, G., Megosh, L. C. and O'brien, T. G. (1996). Modulation of murine hair follicle function by alterations in ornithine decarboxylase activity. *J. Invest. Dermatol.* 106, 1108-1113. 10.1111/1523-1747.ep12340155 8618048

[BIO059647C49] Starks, R., Kirby, P., Ciliberto, M. and Hefti, M. (2018). Snyder-Robinson syndrome. *Autops Case Rep.* 8, e2018031. 10.4322/acr.2018.031 30237987 PMC6140707

[BIO059647C50] Vansickle, E. A., Michael, J., Bachmann, A. S., Rajasekaran, S., Prokop, J. W., Kuzniecky, R., Hofstede, F. C., Steindl, K., Rauch, A., Lipson, M. H. et al. (2021). Expanding the phenotype: Four new cases and hope for treatment in Bachmann-Bupp syndrome. *Am. J. Med. Genet. A* 185, 3485-3493. 10.1002/ajmg.a.62473 34477286 PMC9292803

[BIO059647C51] Wang, F.-W., Guan, X.-Y. and Xie, D. (2013). Roles of eukaryotic initiation factor 5A2 in human cancer. *Int. J. Biol. Sci.* 9, 1013-1020. 10.7150/ijbs.7191 24250246 PMC3831114

[BIO059647C52] Wei, J. H., Cao, J. Z., Zhang, D., Liao, B., Zhong, W. M., Lu, J., Zhao, H. W., Zhang, J. X., Tong, Z. T., Fan, S. et al. (2014). EIF5A2 predicts outcome in localised invasive bladder cancer and promotes bladder cancer cell aggressiveness in vitro and in vivo. *Br. J. Cancer* 110, 1767-1777. 10.1038/bjc.2014.52 24504366 PMC3974079

[BIO059647C53] Yue, F., Cheng, Y., Breschi, A., Vierstra, J., Wu, W., Ryba, T., Sandstrom, R., Ma, Z., Davis, C., Pope, B. D. et al. (2014). A comparative encyclopedia of DNA elements in the mouse genome. *Nature* 515, 355-364. 10.1038/nature13992 25409824 PMC4266106

[BIO059647C54] Zanelli, C. F. and Valentini, S. R. (2007). Is there a role for eIF5A in translation? *Amino Acids* 33, 351-358. 10.1007/s00726-007-0533-0 17578650

[BIO059647C55] Zhou, J., Pang, J., Tripathi, M., Ho, J. P., Widjaja, A. A., Shekeran, S. G., Cook, S. A., Suzuki, A., Diehl, A. M., Petretto, E. et al. (2022). Spermidine-mediated hypusination of translation factor EIF5A improves mitochondrial fatty acid oxidation and prevents non-alcoholic steatohepatitis progression. *Nat. Commun.* 13, 5202. 10.1038/s41467-022-32788-x 36057633 PMC9440896

[BIO059647C56] Ziegler, A., Steindl, K., Hanner, A. S., Kumar Kar, R., Prouteau, C., Boland, A., Deleuze, J. F., Coubes, C., Bezieau, S., Kury, S. et al. (2022). Bi-allelic variants in DOHH, catalyzing the last step of hypusine biosynthesis, are associated with a neurodevelopmental disorder. *Am. J. Hum. Genet.* 109, 1549-1558. 10.1016/j.ajhg.2022.06.010 35858628 PMC9388783

