# Supplementary material for: New K50R mutant mouse models reveal impaired hypusination of eif5a2 with alterations in cell metabolite landscape
Source: Biol Open. 2023 Mar 21;12(3):bio059647. doi: 10.1242/bio.059647 (PMC10084858; doi:10.1242/bio.059647)
Supplement: Supplementary information [file biolopen-12-059647-s1.pdf]

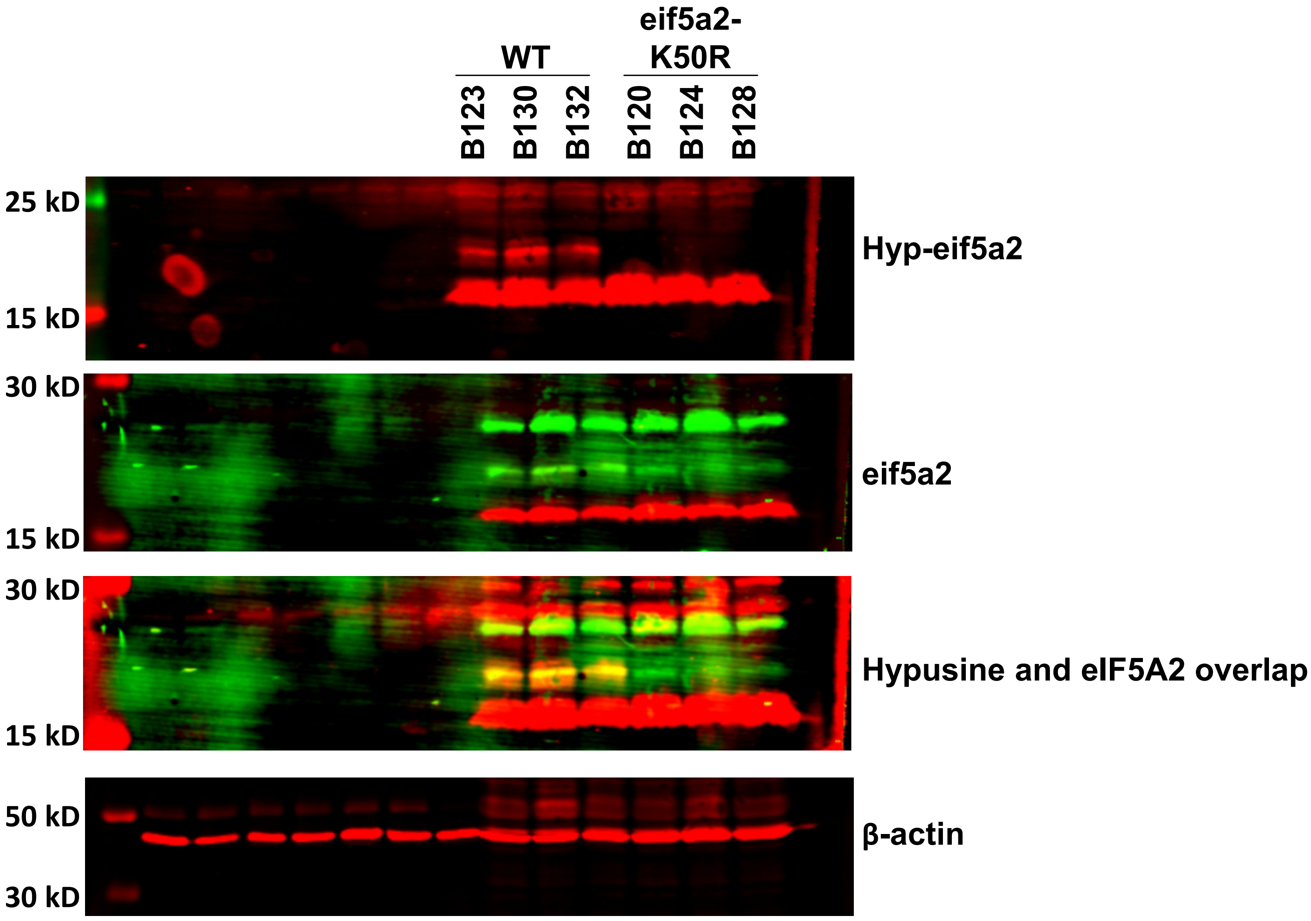


**Fig. S1.** Color representation of Figure 3B of the manuscript confirming the upper hypusine band is Hyp-eIF5A2. Mouse brain lysates were run on a Western blot. Hypusinated eIF5A2 was detected first using a rabbit anti-hypusine antibody followed by a goat anti-rabbit 680 (red) secondary antibody. Without removing the first antibody detection, the blot was then detected for eIF5A2 using a mouse anti-eIF5A2 antibody followed by a goat anti-mouse 800 (green) secondary antibody. The overlap of the upper red band in the hypusine blot with the green eIF5A2 signal (yellow) confirms that the upper band is indeed hypusinated eIF5A2. As expected, this hypusination is missing in the eif5a2-K50R mutants.

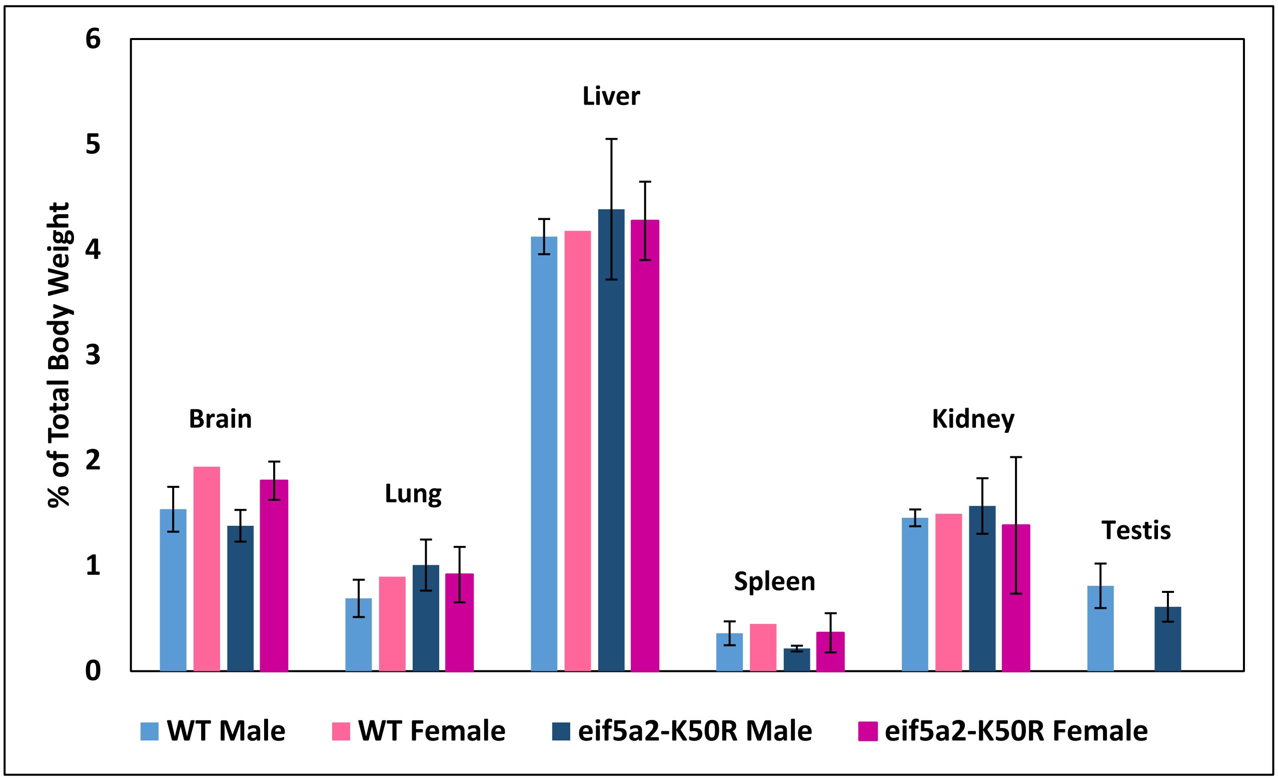


**Fig. S2.** The brain, lungs, liver, spleen, kidneys, and testis of wild-type (WT) and eif5a2-K50R mutant129/Svj mice were harvested and weighed in grams (g). The organ weights were normalized to total mouse weight and graphically represented as % of Total Body Weight. No significant differences in organ weights were noted. Data represents the mean +/- the standard deviation of 1-4 mice per group.

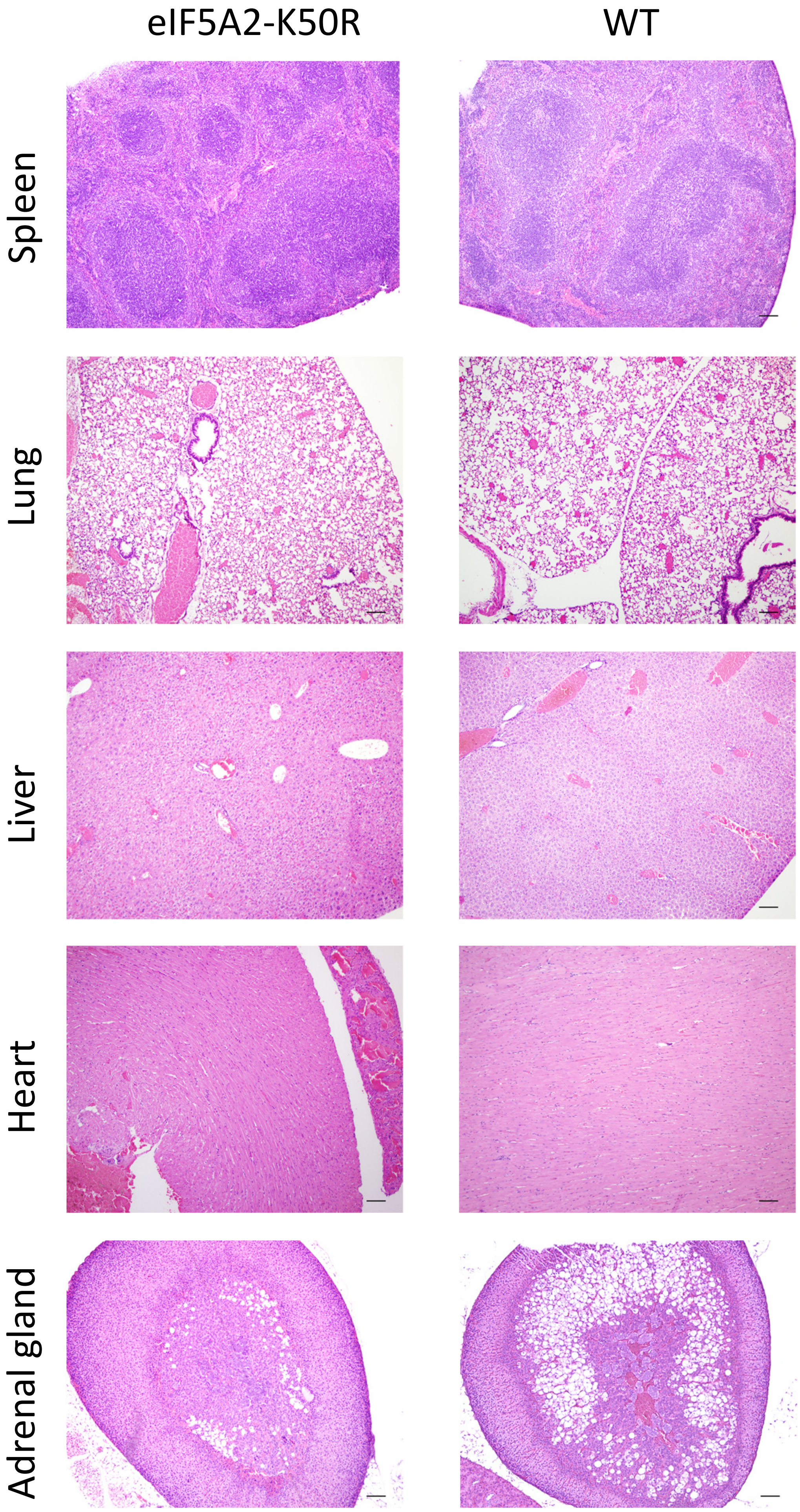
eIF5A2-K50R
WT
Spleen
Lung
Liver
Heart
Adrenal gland

# Supplementary Figure 3 (continued)

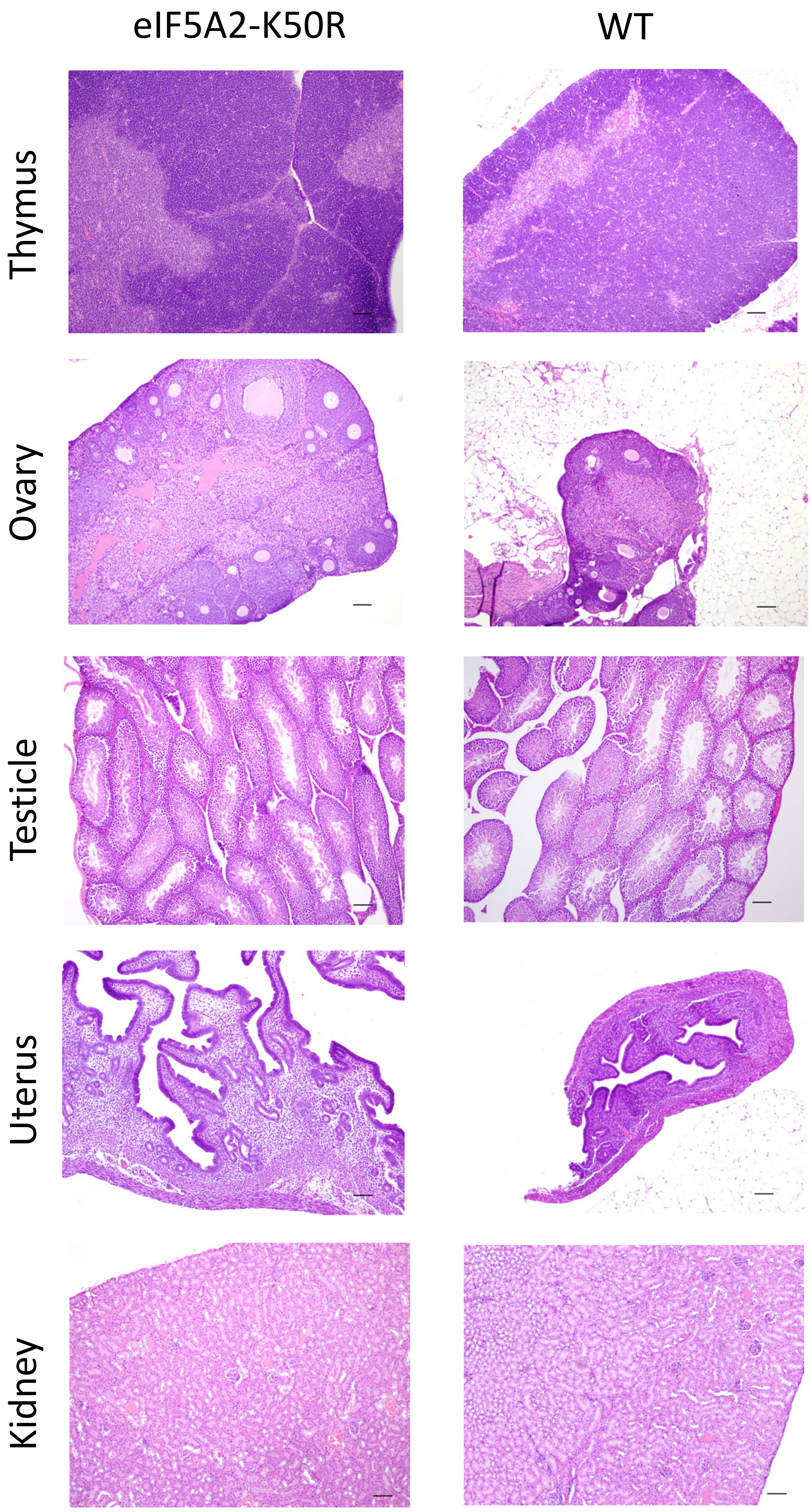

# Supplementary Figure 3 (continued)

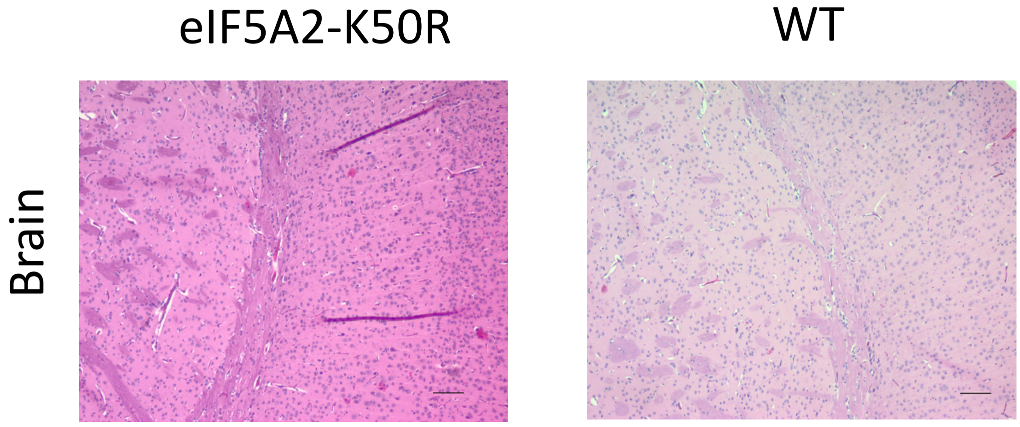


**Fig. S3.** Photomicrographs of representative sections of spleen, lung, liver, heart, adrenal gland, thymus, ovary, testicle, uterus, kidney, and brain from six eIF5A2-K50R and two wild-type mice. No significant differences were noted between the eIF5A2-K50R mice and wild-type (WT) mice. All abnormalities noted were considered background lesions or variations in the stage of the reproductive cycle and not relevant to the study. The variation in the adrenal cortical vacuolation is a common background lesion and was inconsistently seen in either group. HE stain. Bar = 100 µm.

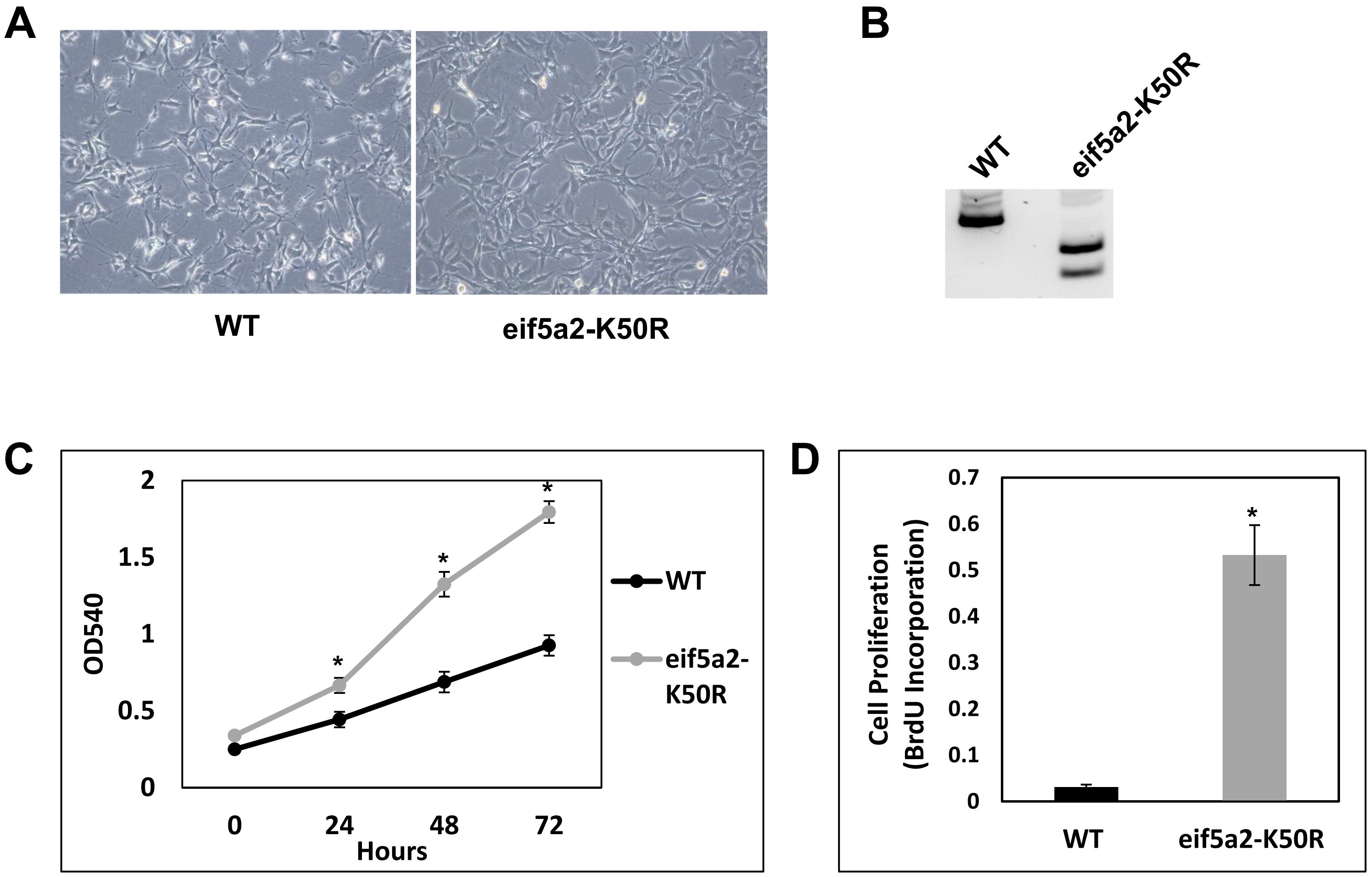


**Fig. S4.** Mouse dermal fibroblasts with mutant eif5a2-K50R proliferate faster than wild-type (WT) fibroblasts. (A) Light micrographs (10X) of dermal fibroblasts derived from both WT and eif5a2-K50R mutant mouse ear punch biopsies. (B) DNA gel image confirming the genotype of the dermal fibroblasts. PCR product containing the K50R mutation is cut by the restriction enzyme PflFI, while WT is not. (C) eif5a2-K50R dermal fibroblasts grow faster than WT fibroblasts as indicated by more intense sulforhodamine B staining as measured by optical density at 540 nm (OD540). Data represents the mean +/- the standard error (S.E.) of three independent experiments done at a minimum of eight replicates per experiment (N=26). (D) eif5a2-K50R dermal fibroblasts proliferate faster than WT fibroblasts as measured by increased BrdU incorporation. Data represents the mean +/- S.E. of three independent experiments with six replicates per experiment (N=18). * denotes a statistically significant difference (p<0.005) between WT and eif5a2-K50R dermal fibroblasts.

Biology Open • Supplementary information

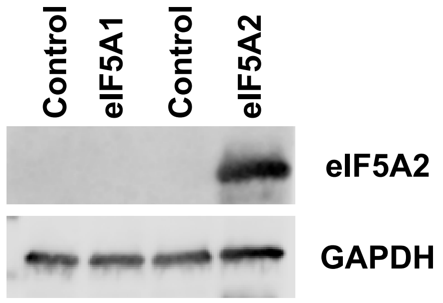


**Fig. S5.** eIF5A2 antibody does not cross react with eIF5A1. Human embryonic kidney 293 (HEK-293) cell lysates that overexpress eIF5A1 (Origene, LC419616) or eIF5A2 (Origene, LC412495) were run on a western blot and detected with mouse anti-eIF5A2 antibody (Origene, TA505100) to test its specificity. The antibody was specific to eIF5A2 and did not cross react with eIF5A1 as is evident by a single band in the eIF5A2 overexpression lysate lane.

Biology Open • Supplementary information
